# Suzuki-Miyaura Reactions Catalyzed by *C_2_*-Symmetric Pd-Multi-Dentate *N*-Heterocyclic Carbene Complexes

**DOI:** 10.3390/molecules171012121

**Published:** 2012-10-16

**Authors:** Lan Jiang, Fengjun Shan, Zhengning Li, Defeng Zhao

**Affiliations:** 1 State Key Laboratory of Fine Chemicals, Dalian University of Technology, Dalian 116024, China; Email: jianglan@dlu.edu.cn; 2 College of Environmental and Chemical Engineering, Dalian University, Dalian 116622, China; Email: sfj225@126.com

**Keywords:** imidazolium salt, *N*-heterocyclic carbene, palladium, Suzuki-Miyaura coupling, catalysis

## Abstract

Suzuki-Miyaura coupling reactions are promoted by Pd complexes ligated with *C_2_*-symmetric multi-dentate *N*-heterocyclic carbenes derived *in situ* from Pd(OAc)_2_ and imidazolium salts. Good to excellent yields were obtained for aryl bromides as substrates. Turnover numbers of up to 10^5^ could be achieved with 5 × 10^−4^ mol% of Pd(OAc)_2_/1 × 10^−3^ mol% NHC precatalyst in 24 h.

## 1. Introduction

The formation of C-C bonds catalyzed by transition metal complexes represents one of the most powerful tools in organic synthesis [1,2,3] and has found important applications in the synthesis of organic molecules such as pharmaceuticals [4], natural products [5], and polymers [6]. The Suzuki-Miyaura reaction, the C-C cross-coupling reaction between aryl halides and arylboronic acids, is an example of this kind of reaction [7,8,9]. As is well known, many Pd-phosphine complexes have been employed as the catalysts for this transformation [10,11], but most phosphine ligands, especially those displaying good catalytic properties, are expensive, toxic and air-sensitive. Accordingly, Pd-complexes overcoming these limitations are highly desirable. *N*-Heterocyclic carbenes (NHCs) have received increasing attention after the isolation of free NHC by Arduengo and co-workers in 1991 [12], and Herrmann’s seminal work demonstrating the catalysis of coupling reactions using Pd-NHC complexes [13]. The excellent σ-donor and lower π-acceptor characteristics of NHC, in combination with their good stability towards air and moisture, make them attractive as ligands in catalytic reactions [14,15,16]. Furthermore, substituents attached to the NHC framework could be easily modulated to tune their electronic as well as the steric properties. These results have shown NHCs to be an alternative for conventional phosphine ligands in homogeneous catalysis including olefin metathesis [17], hydrosilylation [18,19], hydrogenation [20,21], C-C coupling reactions, *etc*. [22].

**Figure 1:**
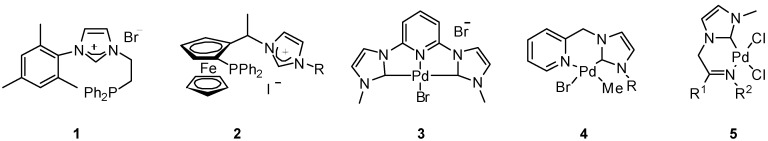
Typical Pd-NHC complexes and NHC precursors with a (potentially) coordinative heteroatom.

Suzuki-Miyaura coupling reactions promoted by Pd-NHC complexes were initiated by Herrmann’s report in 1998 [23]. Since then, Pd-NHC complexes have been found as efficient catalysts for this kind of coupling reaction [24,25,26,27,28,29,30], and new NHCs or their precursors have been synthesized to confer more efficient catalytic properties or ease of operation [31,32,33,34]. Among these catalysts, most of them are derived from monodentated NHCs, and some are bidentate anionic ligands [26,27]. The chelating NHC ligands predominantly consist of two NHC moieties linked by a chain or a ring, e.g., Shi’s *cis*-chelating, bidentate NHC derived from binaphthyl-2,2′-diamine [35], and more importantly, hybrid NHC ligands. Typical examples of the hybrid NHC ligands used in palladium-catalyzed reactions include *NHC*,*P* chelating ligands derived from **1** and **2**, and *NHC*,*N* chelating ligands in complexes **3**, **4** and **5** (Figure 1) [36,37,38,39]. Nonetheless, little attention has been paid to hybrid NHC chelating ligands bearing weakly-coordinating *O*-atom as a potential coordination atom until now [40,41,42].

The fact that Pd complexes containing both NHC and phosphine ligands, show higher activity than those with phosphine as the only ligand or Pd(NHC)_2_X_2_, was reasoned to be due to the strong Pd-NHC bond and relatively weak Pd-P bond, which results in the stabilization of Pd, easy dissociation of phosphine ligand and favorable oxidative addition of aryl halides to Pd. In metal-NHC complexes promoted reactions, metal complexes with both NHC and phosphine structural motif generally exhibit better activity and selectivity than those only with monodentate NHC ligands. Therefore, a second, relatively weak coordination atom in the chelating metal-NHC complex might be beneficial for improving the catalytic performance.

**Figure 2:**
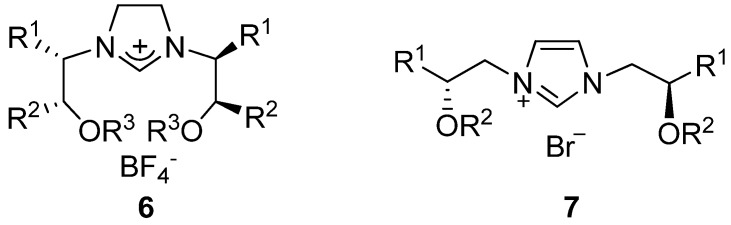
The structure of tridentate *C_2_*-symmetric imidazol(in)ium salts **6** and **7**.

We have an interest in the applications of multidentate, *C_2_*-symmetric NHCs with one chelating carbene and two *O*- atoms as ligands in catalytic reactions, for which there have been few precedents. We envisioned that the coordinative ability of the heteroatom can be adjusted by changing its bonding. Previously, we have synthesized tridentate *C_2_*-symmetric imidazolinium salts **6** (Figure 2) and their NHC precursors, which have two oxygen atoms in the arms, and found that their copper complexes could catalyze the asymmetric conjugate addition of Et_2_Zn to cycloalkenones efficiently [43]. To continue this work, we designed **7**, the unsaturated counterpart of **6**, to explore their applications in Suzuki-Miyaura coupling reactions, as it has been reported that Pd coordinated with unsaturated NHCs show higher reactivity than those with saturated NHCs [44]. Herein, we wish to report the preparation and catalytic properties of the novel *C_2_*-symmetric tridentate imidazolium salts **7** in the Suzuki-Miyaura coupling reaction.

## 2. Results and Discussion

**Scheme 1:**
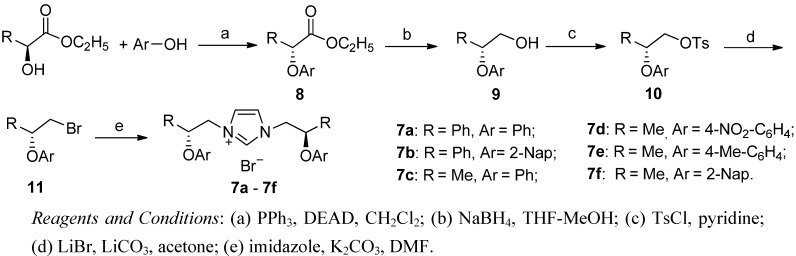
Synthesis of the NHC precursor imidazolium salts **7a–f**.

The synthesis of the NHC precursor imidazolium salts **7a–f** in 22-30% overall yield is shown in Scheme 1. The synthetic route to the NHC precursor imidazolium salts **7a–f** started from (*S*)-ethyl lactate or (*S*)-ethyl mandelate by etherification with phenol or substituted phenols. The *α*-aryloxycarboxylates **8a–f** were next reduced, yielding *β*-aryloxyalcohols **9a–f**. Then the alcohol were converted into halides **11a–f**, and reacted with imidazole giving imidazolium salts **7a–f**, which contain *β*-aryloxyl groups in the side chains. They are quite stable towards air and moisture.

The imidazolium salts **7c–f** synthesized from (*S*)-ethyl lactate were optically pure. This can be deduced from the fact that only one set of NMR signals was observed in both ^1^H-NMR and ^13^C-NMR spectra of these compounds. Reaction of racemic bromides **11c–f** with imidazole would yield racemic *C_2_*-symmetric (*R,R*), (*S,S*)-isomers of **7c–f**, and *Cs*-symmetric meso-(*R*,*S*)-isomer of **7c–f**. Even though the (*R,R*), (*S,S*)-isomers could not be distinguished by NMR, the signals of the racemic isomers and *meso*-isomer should be different. Therefore, the sets of NMR signals of **7** could be used to judge whether the products are optically pure.

On the other hand, use of (*S*)-ethyl mandelate yielded **7a** and **7b** as a mixture of diastereomers, since ^1^H-NMR and ^13^C-NMR signals for two sets or more than one set were observed. This indicates that racemization occurs using (*S*)-ethyl mandelate as the starting material, whose α-H is more acidic than that in ethyl lactate.

The catalytic activities of the Pd-complexes of the NHCs derived from imidazolium salts **7** in Suzuki-Miyaura coupling reactions were screened, using catalysts generated *in situ* from a mixture in a solvent of **7**, Pd(OAc)_2_, and a base.

The reaction conditions were optimized on a model reaction between phenyl bromide and phenylboronic acid. First, the effect of solvents was examined using the catalyst generated from imidazolium salt **7a** and Pd(OAc)_2_. The product biphenyl **12a** was obtained in 95% yield in either toluene or DMF. Addition of H_2_O to DMF (V/V = 1:2) accelerated the reaction but no increase in the yield was observed. The reaction was completed in 1.5 h using toluene as the solvent. Moderate to low yields were obtained when polar solvents, including 1,4-dioxane, THF and EtOH, were used, even though 1,4-dioxane and EtOH have been reported in the literature as good solvents for this kind of reaction. 

Using toluene as the solvent, bases were then optimized, and the results are shown in Table 1. Although Cs_2_CO_3_ is generally efficient for the Suzuki-Miyaura coupling reactions catalyzed by Pd-NHC or palladium-phosphine complexes, in our case, only a 32% yield of **12a** was observed, whereas the use of K_2_CO_3_ resulted in an excellent yield (entry 2). With other bases like NaOH, KF and K_3_PO_4_, the reaction also proceeded well, but somewhat more slowly. Then the temperature was optimized. Phenyl bromide was quantitatively converted to **12a** in 0.5 h at 110 °C, much faster than at 90 °C. At lower temperature, the reaction is very slow. Only a 53% yield of **12a** was obtained after 21 h at 70 °C. Therefore, subsequent reactions were carried out using K_2_CO_3_ as the base and toluene as the solvent at 110 °C.

**Table 1:** Influence of the base onthe Suzuki-Miyaura reaction catalyzed by Pd(OAc)_2_/**7a** ^a^.

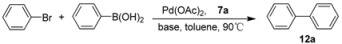			
Entry	Base	Time (h)	Yield ^b^(%)
1	Na_2_CO_3_	24	12
2	K_2_CO_3_	1.5	95
3	Cs_2_CO_3_	24	32
4	NaOH	24	86
5	KF	24	75
6	K_3_PO_4_	24	82
7	CH_3_COONa	24	4

^a^ *Reaction conditions*: 0.5 mmol phenyl bromide, 0.75 mmol phenylboronic acid, 1.5 mmol base, 0.5 mol% Pd(OAc)_2_, 1 mol% **7a**, 3.0 mL toluene, 90 °C. ^b^ GC yield.

Next, the catalytic abilities of **7a** analogues were evaluated and the results are shown in Table 2. The most efficient catalyst was derived from **7a** (entry 1). A comparable yield was obtained with the methyl substituted analogue **7c** (entry 3). Introduction of a nitro group to the aryloxy substituent results in a ligand-Pd complex generating a lower yield of the coupling product (entry 4), which shows that reduction of the electron density of the phenyl ring and the resultant decrease in the coordination ability of the *O-*atom reduced the catalytic ability of **7**. The presence of a 4-methyl group in the aryloxy substituent also led to a lower yield (entry 5), but higher than that using **7d**. Yields of 86–87% were generated when the (substituted)-phenoxy substituent was changed to a 2-naphthoxy group (entries 2 and 6), which is more steric demanding and resulted in the *O-*atom being less coordinative. Two typical imidazolium salts IMes·HCl (IMes = 1,3-bis(2,4,6-trimethylphenyl)-imidazol-2-ylidene) and IPr·HCl (IPr = 1,3-bis(2,6-diisopropylphenyl)-imidazol-2-ylidene) catalyze the coupling reaction giving inferior results (entries 7, 8). In the absence of imidazolium salt or other ligand, a 72% yield of **12a** was obtained in 1h using Pd(OAc)_2_ as the catalyst (entry 9). These results demonstrate that the ligand derived from imidazolium salts **7** which contains two potentially chelated oxygen atoms is more efficient than simple NHC ligands, e.g., IPr and IMes in the coupling reactions. This might be due to the presence of a NHC moiety and oxygen atoms in the hybrid NHC ligands. The strong coordinating of NHC can prevent the dissociate of ligand from palladium complex, and therefore, stablize the catalytically active palladium species. In the meanwhile, the weak coordinative oxygen atom(s) can easily dissociate from palladium complex and generate a coordination-unsaturated palladium species. This favors the oxidation of aryl halides or arylboronic acids toward the coordination-unsaturated palladium species and catalytic cycle. Indeed, formation of palladium balck, which is catalytic inert to the Suzuki reaction and an indication of ligands dissociate from palladium completely, is rarely observed in the coupling reaction using **7**. The coupling reaction of phenylboric acid with phenyl bromide could be performed in air with a slightly reduced yield (78%).

**Table 2:** Evaluation of the NHCsfrom **7a–f** in the Suzuki-Miyaura reaction ^a^.

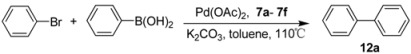			
Entry	Imidazolium salt	Time (h)	Yield *^b^*(%)
1	**7a**	0.5	99
2	**7b**	1	87
3	**7c**	1	96
4	**7d**	1	85
5	**7e**	1	90
6	**7f**	1	86
7	IPr·HCl	1	75
8	IMes·HCl	1	70
9	-	1	72

^a^ *Reaction conditions*: 0.5 mmol phenyl bromide, 0.75 mmol phenylboronic acid, 1.5 mmol K_2_CO_3_, 0.5 mol% Pd(OAc)_2_, 1 mol% **7**, 3.0 mL toluene, 110 °C. ^b^ GC yield.

The reactions of various aryl halides with phenylboronic acids were then investigated under the optimized conditions, and the results are summarized in Table 3. Only 5 minutes were needed for the complete reactions of 4-nitrophenyl bromide and 4-acetophenyl bromide, which are electron-deficient and more reactive in this kind of reaction.

**Table 3:** Suzuki-Miyaura reaction of aryl halides (benzyl halides) with phenylboronic acid catalyzed by Pd(OAc)_2_/**7a**.

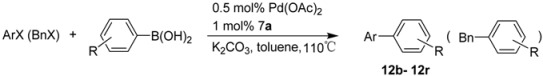					
Entry	ArX or BnX	R	Time	Product	Yield ^a^ (%)
1	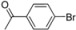	H	5 min	**12b**	95
2	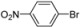	H	5 min	**12c**	98
3	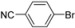	H	1 h	**12d**	98
4	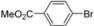	H	0.5 h	**12e**	99
5	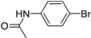	H	0.5 h	**12f**	96
6	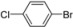	H	1 h	**12g**	93
7	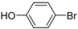	H	0.5 h	**12h**	95
8	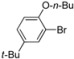	H	1 h	**12i**	92
9	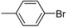	H	0.5 h	**12j**	91
10	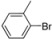	H	1 h	**12k**	85
11	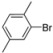	H	1 h	**12l**	90
12	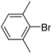	H	1 h	**12m**	96
13	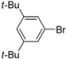	H	1 h	**12n**	97
14	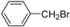	H	3 h	**12o**	90
15	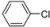	H	24 h	**12a**	35
16	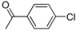	H	24 h	**12b**	70
17	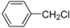	H	10 h	**12o**	71
18	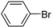	3-Me	0.5 h	**12p**	93
19	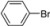	4-Me	0.5 h	**12j**	85
20	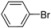	2-Me	2.5 h	**12k**	93
21	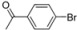	4-MeO_2_C	24 h	**12q**	86
22	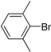	2-Me	1 h	**12r**	21 *^b^*
23	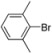	2-Me	11.5 h	**12r**	56 *^b,c^*

*^a^* Isolated yield except noted. *^b^* GC yield. *^c^* Using 0.05 mol% Pd(OAc)_2_, 0.1 mol% imidazolium salt **7a**.

For those aryl halides with electron-donating groups (entries 7, 10, 13) or sterically hindered aryl bromides (entry 11), longer reaction times were needed. Generally, almost quantitative yields of coupling products could be obtained in 1 h. It is worth to note that 4-hydroxylphenyl bromide, which is very electron-rich in the phenyl ring and the C-Br bond, is highly reactive to coupling. As expected, a wide range of functional groups including keto, nitro, cyano, ester, amide, hydroxy and ether, were tolerated. The coupling reactions could be extended to benzyl bromide which contains a sp^3^-C atom bearing the halide, and therefore, a C-C coupling product between a sp^3^-C and a sp^2^-C was achieved (entry 14). The reaction of 4-chlorophenyl bromide gave 4-chlorobiphenyl as the only coupling product in high yield, and demonstrated the inertness of C-Cl in the presence of C-Br (entry 6). When phenyl chloride was used, only a 35% yield of biphenyl was obtained (entry 15). Introduction of an acetyl group to phenyl chloride led to an increase in the activity, and a 70% yield of the coupling product was achieved (entry 16). Gratifyingly, benzyl chloride also gave a reasonable yield of coupling product (entry 17), although in lower yield than that obtained with benzyl bromide. Palladium black was only observed unremarkably in reactions performed at 110 °C and after long reaction times.

In addition, reactions of various arylboronic acids with substituted phenyl bromides were performed. The reactions proceed smoothly in high yield using 2-tolylboronic acid. In contrast to aryl halides, the presence of an electron-withdrawing group results in a low coupling yield which may be attributed to the tendency of hydrolysis of the electron deficient arylboronic acids (Table 3, entry 21) [45]. Even though the presence of an *ortho-*Me group in either the aryl halide or the arylboronic acid did not affect the coupling yield, it was still difficult to obtain tri*-ortho* substituted biphenyl in high yields, a challenging reaction in literature, due to the large hindrance for our catalyst system. In the case of a very sterically hindered reaction (entry 22), the less bulky 2,2′-dimethylbiphenyl (the homocoupling product of two arylboronic acid molecules), was formed in preference to the more bulky 2,6,2′-trimethylbiphenyl product derived from the expected Suzuki-Miyaura coupling when 0.5 mol% of Pd catalyst was employed. This indicated the relative inertness of the 2,6-dimethylphenyl bromide. Therefore, the amount of the Pd salt was decreased to 0.05 mol% to depress the relative faster coupling reaction between the aryl groups in the boronic acid. In this case, the yield of the trimethylbiphenyl increased to 56% as judged by gas chromatography over a prolonged reaction time (entry 23). Similar results were observed for the reaction of 4-bromoacetophenone with phenylboronic acid (entries 1, 2 in Table 4 *vs*. entry 1 in Table 3).

**Table 4:** The activities of Pd(OAc)_2_/**7a** with variation of loading.

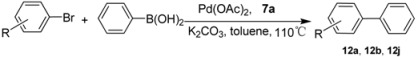						
Entry	R	Pd (mol%)	Time(h)	Product	Yield ^a^ (%)	TON
1	COMe	0.05	1	**12b**	100	2000
2	COMe	0.005	4	**12b**	100	20000
3	COMe	0.0005	24	**12b**	50	100000
4	H	0.05	2	**12a**	95	1900
5	H	0.005	2	**12a**	38	7600
6	Me	0.05	1.5	**12j**	100	2000
7	Me	0.005	4	**12j**	95	19000

^a^ GC yield using diethylene glycol di-*n*-butyl ether as an internal standard.

**Figure 3:**
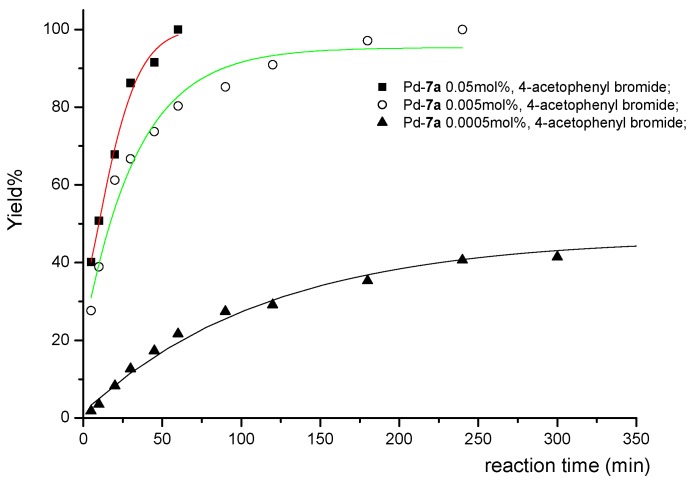
Reaction profiles for Suzuki-Miyaura reaction between 4-acetylphenyl bromide and phenylboronic acid catalyzed by different Pd/**7a** loading: 0.05 mol% (■), 0.005 mol% (○), 0.0005 mol% (▲).

**Figure 4:**
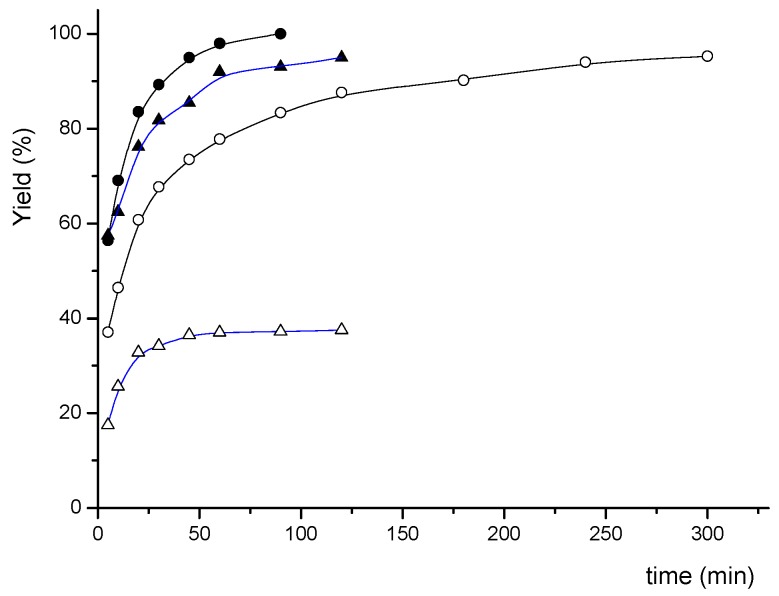
Reaction profiles for Suzuki-Miyaura reaction of 4-methylphenyl bromide catalyzed by Pd/**7a** 0.05 mol% (●), 0.005 mol% (○), and phenyl bromide catalyzed by Pd/**7a** 0.05 mol% (▲), 0.0005 mol% (△).

The efficiencies of Pd(OAc)_2_/**7a** were further tested with different catalyst loadings, and the results are summarized in Table 4. 4-Acetylphenyl bromide could be completely converted to the coupling product using 5 × 10^−2^ mol% in 1 h and 5 × 10^−3^ mol% of Pd catalyst in 4 h, respectively (entries 1 and 2). The turnover numbers (TON) reached 2 × 10^4^. Furthermore, a maximum TON of 10^5^ was obtained at 5 × 10^−4^ mol% of catalyst loading in 24 h (entry 3), and the turnover frequency (TOF ) is *ca.* 4.17 × 10^3^ h^−1^. A TON of up to 7,600 was recorded for the reaction of phenyl bromide (entry 5). Interestingly, for the less active 4-bromotoluene, TON and TOF values up to 1.9 × 10^4^ and 4.75 × 10^3^ h^−1^, respectively, could be achieved with 5 × 10^−3^ mol% Pd catalyst in 4 h (entry 7), which was better than the case of phenyl bromide. Details of the progress of reactions with different amount of catalyst and substrates are given in Figure 3 and Figure 4.

## 3. Experimental

### 3.1. General

All chemicals were purchased from Alfa Aesar Co., Ltd. (Tianjin, China) and Accela ChemBio Co., Ltd. (Shanghai, China), except arylboronic acids which were products of Ally Chemical Ltd. (Dalian, China). The solvents were freshly distilled prior to use. NMR spectra were recorded on a Varian 400 MHz spectrometer or on a Bruker DRX500 spectrometer, using TMS as an internal standard. IR spectra were recorded on a Nicolet 550 spectrometer. MS spectra were measured on a Hewlett-Packard HP-6890/5973 gas chromatography-mass spectrometer. HRMS were recorded on a Micromass UPLC/Q-Tof Micro spectrometer. The reaction mixtures were analyzed by gas chromatography (Shimadzu GC-2010, capillary column SE-54, 30 m × 0.32 mm × 4 μm; FID detector; N_2_ gas). Column chromatography was performed with silica gel (200–300 mesh).

### 3.2. Synthesis of Imidazolium Salts ***7a–f*** (Exemplified by the Synthesis of ***7f***)

#### 3.2.1. Ethyl 2-(2-Naphthoxyl)propanoate (**8f**)

*β*-Naphthol (1.586 g, 11.00 mmol), PPh_3_ (3.148 g, 12.00 mmol) and dichloromethane (10 mL) were added to a round-bottom flask, and the mixture was stirred for 5 min. (*S*)-Ethyl lactate (1.182 g, 10.00 mmol) was added to the flask, then the flask was cooled with an ice-water bath. DEAD (1.742 g, 10.00 mmol) was added slowly. The mixture was stirred for 30 min at 0 °C, and then 6 h at room temperature. After removal of the colorless precipitate by filtration, the filtrate was concentrated *in vacuo*. **8f** (2.230 g, 91.2% yield) was isolated by column chromatography as colorless oil. R_f_ = 0.57 (EA: PE = 10: 90), 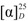 = +12.1 (*c* 1.46, EtOH). ^1^H-NMR (400 MHz, CDCl_3_) δ 7.72 (d, *J* = 9.0 Hz, 2H, Ar-*H*), 7.67 (d, *J* = 8.2 Hz, 1H, Ar-*H*), 7.40 (ddd, *J* = 8.2, 6.9, 1.2 Hz, 1H, Ar-*H*), 7.31 (ddd, *J* = 8.1, 6.9, 1.2 Hz, 1H, Ar-*H*), 7.19 (dd, *J* = 8.9, 2.6 Hz, 1H, Ar-*H*), 7.04 (d, *J* = 2.5 Hz, 1H, Ar-*H*), 4.87 (q, *J* = 6.8 Hz, 1H, C*H*), 4.27–4.13 (m, 2H, C*H*_2_), 1.66 (d, *J* = 6.8 Hz, 3H, C*H*_3_CH), 1.21 (t, *J* = 7.1 Hz, 3H, C*H*_3_CH_2_). ^13^C-NMR (101 MHz, CDCl_3_) δ 171.99 (*C*=O), 155.44, 134.21, 129.55, 129.22, 127.52, 126.74, 126.31, 123.85, 118.78, 107.60 (Ar-*C*), 72.51 (*C*H), 61.10 (*C*H_2_), 18.40 (*C*H_3_CH), 13.99 (*C*H_3_CH_2_).

#### 3.2.2. 2-(2-Naphthoxyl)propan-1-ol (**9f**)

To an ice-water cooled round-bottom flask containing a solution of **8f** (1.243 g, 5.09 mmol) in THF (10.0 mL) was added NaBH_4_ (0.380 g, 10 mmol) in portions, and the mixture was stirred for 30 min at 0 °C. Then the temperature was recovered to room temperature slowly, and the mixture was stirred at room temperature for 6 h. The volatiles were removed by evaporation *in vacuo*. Water was added to the residue, and the aqueous phase was extracted with dichloromethane. The combined organic phase was dried with Na_2_SO_4_ and purified by column chromatography, yielding **9f** (0.85 g, 84.2% yield) as colorless oily liquid. R_f_ = 0.14 (EA:PE = 10:90), 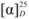 = −14.5 (*c* 1.11, EtOH). ^1^H-NMR (400 MHz, CDCl_3_) δ 7.75–7.61 (m, 3H, Ar-*H*), 7.39 (dd, *J* = 8.1, 7.0 Hz, 1H, Ar-*H*), 7.30 (dd, *J* = 8.0, 7.0 Hz, 1H, Ar-*H*), 7.15 (d, *J* = 2.1 Hz, 1H, Ar-*H*), 7.11 (dd, *J* = 8.9, 2.4 Hz, 1H, Ar-*H*), 4.61–4.51 (m, 1H, C*H*), 3.77–3.69 (m, 2H, C*H*_2_), 2.88 (s, 1H, O*H*), 1.25 (d, *J* = 6.2 Hz, 3H, C*H*_3_). ^13^C-NMR (101 MHz, CDCl_3_) δ 155.50, 134.51, 129.61, 129.13, 127.64, 126.78, 126.42, 123.83, 119.51, 108.98 (Ar-*C*), 74.75 (*C*H), 66.11 (*C*H_2_), 15.73 (*C*H_3_).

#### 3.2.3. 2-(2-Naphthoxyl)propyl 4-methylbenzenesulfonate (**10f**)

To a round-bottom flask, **9f** (0.850g, 3.93 mmol) and dichloromethane (15 mL) was added, followed by tolylsulfonyl chloride (0.964 g, 5.06 mmol) and pyridine (0.396 g, 5.01 mmol). The mixture was stirred at room temperature for 24 h, and quenched by the addition of water. After phases separation and general workup, oily **10f** (1.104g, 73.8% yield) was isolated. R_f_ = 0.28 (EA:PE = 20:80), 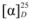 = +70.27 (*c* 1.04, CHCl_3_). ^1^H-NMR (400 MHz, CDCl_3_) δ 7.73 (t, *J* = 7.1 Hz, 3H, Ar-*H*), 7.65 (dd, *J* = 13.7, 8.6 Hz, 2H, Ar-*H*), 7.41 (t, *J* = 7.5 Hz, 1H, Ar-*H*), 7.31 (t, *J* = 7.5 Hz, 1H, Ar-*H*), 7.19 (d, *J* = 8.0 Hz, 2H, Ar-*H*), 7.01 (s, 1H, Ar-*H*), 6.98 (dd, *J* = 8.9, 2.4 Hz, 1H, Ar-*H*), 4.72–4.63 (m, 1H, C*H*), 4.20 (dd, *J* = 10.6, 5,8 Hz, 1H, CH-*H*), 4.14 (dd, *J* = 10.6, 4.4 Hz, 1H, CH-*H*), 2.31 (s, 3H, Ph-C*H*_3_), 1.33 (d, *J* = 6.3 Hz, 3H, C*H*_3_CH). ^13^C-NMR (101 MHz, CDCl_3_) δ 154.95, 144.93, 134.36, 132.82, 129.84, 129.58, 129.21, 127.90, 127.63, 126.81, 126.47, 123.98, 119.34, 108.76 (Ar-*C*), 71.74 (*C*H), 71.23 (*C*H_2_), 21.55 (Ph-*C*H_3_), 16.44 (*C*H_3_CH).

#### 3.2.4. 1-Bromo-2-(2-naphthoxyl)propane (**11f**)

Compound **10f** (1.10 g, 2.97 mmol), acetone (15 mL), LiBr (0.348 g, 4.01 mmol) and Li_2_CO_3_ (0.030 g, 0.45 mmol) were added to a flask in sequence. The mixture was refluxed for 48 h. After cooled to room temperature, the solvent was removed by evaporation *in vacuo*. The residue was separated by column chromatography, affording oily **11f** (0.654 g, 82.6% yield). R_f_ = 0.69 (EA:PE = 20:80), 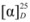 = −41.86 (*c* 1.01, CHCl_3_). ^1^H-NMR (400 MHz, CDCl_3_) δ 7.76 (dd, *J* = 8.3, 2.9 Hz, 2H, Ar-*H*), 7.72 (d, *J* = 8.3 Hz, 1H, Ar-*H*), 7.44 (ddd, *J* = 8.2, 6.9, 1.3 Hz, 1H, Ar-*H*), 7.35 (ddd, *J* = 8.1, 6.9, 1.2 Hz, 1H, Ar-*H*), 7.20–7.12 (m, 2H, Ar-*H*), 4.77–4.66 (m, 1H, C*H*), 3.62 (dd, *J* = 10.4, 4.8 Hz, 1H, CH-*H*), 3.48 (dd, *J* = 10.4, 6.2 Hz, 1H, CH-*H*), 1.52 (d, *J* = 6.1 Hz, 3H, C*H*_3_). ^13^C-NMR (101 MHz, CDCl_3_) δ 155.16, 134.50, 129.83, 129.38, 127.74, 126.89, 126.56, 124.08, 119.60, 109.17 (Ar-*C*), 73.50 (*C*H), 35.43 (*C*H_2_), 18.84 (*C*H_3_).

#### 3.2.5. 1,3-Bis-[2-(2-naphthoxy)propyl]imidazolium Bromide (**7f**)

To a flask, **11f** (0.653 g, 2.46 mmol), DMF (10 mL), imidazole (0.0816 g, 1.20 mmol) and K_2_CO_3_ (0.0552 g, 0.40 mmol) were added. The mixture was refluxed for 48 h, and quenched by the addition of water after cooled to room temperature. The separated aqueous phase was extracted with dichloromethane (3 × 10 mL). The combined organic phase was dried and concentrated. Oily *1,3-bis-[2-(2-naphthoxy)propyl]imidazolium bromide* (**7f**) (0.400 g, 64.4%) was isolated by column chromatography. Overall yield 30.1%. Yellow oil. 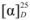 = +41.2 (*c* 0.566, CHCl_3_). IR: υ_C=N_ 1602 cm^−1^, υ_C-O_ 1179 cm^−1^. ^1^H-NMR (400 MHz, CDCl_3_) δ 10.57 (s, 1H, N-C*H*=N), 7.71–7.61 (m, 6H, Ar-*H*), 7.50 (s, 2H, N-C*H*), 7.46–7.38 (m, 2H, Ar-*H*), 7.34 (dd, *J* = 11.0, 4.0 Hz, 2H, Ar-*H*), 7.13 (d, *J* = 2.2 Hz, 2H, Ar-*H*), 7.00 (dd, *J* = 8.9, 2.5 Hz, 2H, Ar-*H*), 5.01–4.96 (m, 2H, O-C*H*), 4.95 (dd, *J* = 15.0, 2.3 Hz, 2H, NCH-*H*), 4.44 (dd, *J* = 14.3, 8.1 Hz, 2H, NCH-*H*), 1.41 (d, *J* = 6.2 Hz, 6H, C*H*_3_). ^13^C-NMR (101 MHz, CDCl_3_) δ 153.80 (N-*C*H=N), 137.18 (N-*C*H=CH), 133.86, 129.50, 128.90, 127.23, 126.50, 126.24, 123.85, 122.79, 118.53, 108.88 (Ar-*C*), 72.07 (O-*C*H), 53.70 (*C*H_2_), 16.25 (C*H*_3_). HRMS: *m/z* 437.2233 (calculated 437.2229 for C_29_H_29_N_2_O_2_).

#### 3.2.6. Compounds ***7a–e*** were Similarly Prepared and Characterized

*1,3-Bis-(2-phenoxy-2-phenylethyl)imidazolium Bromide* (**7a**). Total yield 23.6%. Yellow solid. IR: υ_C=N_ 1602 cm^−1^, υ_C-O_ 1172 cm^−1^. ^1^H-NMR (500 MHz, CDCl_3_) δ 10.78 (s, 1H, N-C*H*=N), 10.77 (s, 1H, N-C*H*′=N), 7.51–7.45 (m, 8H, Ar-*H*), 7. 38 (d, *J* = 1.5 Hz, 2H, N-C*H*), 7.34 (d, *J* = 1.5 Hz, 2H, N-C*H*’), 7.33–7.27 (m, 12H, Ar-*H*), 7.18–7.10 (m, 8H, Ar-*H*), 6.92–6.86 (m, 4H, Ar-*H*), 6.78–6.77 (m, 8H, Ar-*H*), 5.72 (dd, *J* = 8.6, 2.8 Hz, 1H, O-C*H*), 5.64 (dd, *J* = 8.4, 2.8 Hz, 1H, O-C*H′*), 4.93 (dd, *J* = 14.3, 2.9 Hz, 1H, NCH-*H*), 4.92 (dd, *J* = 14.2, 2.9 Hz, 1H, NCH-*H*′), 4.51 (dd, *J* = 14.3, 8.2 Hz, 2H, NCH-*H*), 4.49 (dd, *J* = 14.2, 8.4 Hz, 2H, NCH-*H*′). ^13^C-NMR (101 MHz, CDCl_3_) δ 156.53 (N-*C*H=N), 156.49 (N-*C*′H=N), 138.98 (N-*C*H=CH), 138.78 (N-*C*′H=CH), 135.85, 135.72, 129.65, 129.64, 129.21, 129.05, 129.02, 126.29, 126.17, 122.55, 122.50, 122.02, 122.00, 115.82, 115.72 (Ar-*C*), 78.03 (O-*C*H), 77.91 (O-*C*′H), 55.51 (*C*H_2_). HRMS: *m/z* 461.2222 (calcd. 461.2229 for C_31_H_29_N_2_O_2_). Two sets of NMR signals indicated the racemization of **7a**.

*1,3-Bis-[2-(2-naphthoxy)-2-phenylethyl]imidazolium Bromide*(**7b**). Total yield 28.4%. Yellow oil. 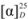 = −9.80 (*c* 0.21, CHCl_3_). IR: υ_C=N_ 1602 cm^−1^, υ_C-O_ 1179 cm^−1^. ^1^H-NMR (400 MHz, CDCl_3_) δ 10.29 (s, 1H, N-C*H*=N), 7.61–7.50 (m, 5H, Ar-*H*), 7.50–7.38 (m, 7H, Ar-*H*, N-C*H*), 7.30–7.11 (m, 10H, Ar-*H*), 7.06 (ddd, *J* = 16.6, 9.0, 2.1 Hz, 2H, Ar-*H*), 6.95 (s, 2H, Ar-*H*), 5.87 (d, *J* = 7.9 Hz, 1H, NCH-*H*), 5.80 (d, *J* = 7.5 Hz, 1H, NCH-*H*), 4.97–4.77 (m, 2H, O-C*H*), 4.52 (d, *J* = 13.7, 8.2 Hz, 1H, NCH-*H*), 4.50 (d, *J* = 13.0, 8.4 Hz, 1H, NCH-*H*). ^13^C-NMR (101 MHz, CDCl_3_) δ 154.16 (N-*C*H=N), 137.81 (N-*C*H=CH), 135.72, 135.61, 133.85, 129.48, 129.03, 128.90, 128.73, 127.37, 126.77, 126.28, 126.16, 124.01, 122.72, 118.44, 118.36, 109.45 (Ar-*C*), 77.47 (O-*C*H), 55.04 (*C*H_2_). HRMS: *m/z* 561.2527 (calcd. 561.2542 for C_39_H_33_N_2_O_2_). More than one set of NMR signals indicated the racemization of **7b**.

*1,3-Bis-(2-phenoxypropyl)imidazolium Bromide* (**7c**). Total yield 21.8%. Yellow oil. 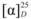 = −20.55 (*c* 0.69, CHCl_3_). IR: υ_C=N_ 1602 cm^−1^, υ_C-O_ 1172 cm^−1^. ^1^H-NMR (400 MHz, CDCl_3_) δ 10.26 (s, 1H, N-C*H*=N), 7.63 (s, 2H, N-C*H*), 7.21 (t, *J* = 7.7 Hz, 4H, Ph-*H*), 6.94 (t, *J* = 7.2 Hz, 2H, Ph-*H*), 6.82 (d, *J* = 8.1 Hz, 4H, Ph-*H*), 4.89 (d, *J* = 14.0 Hz, 2H, NCH-*H*), 4.85–4.79 (m, 2H, O-C*H*), 4.44 (dd, *J* = 14.0, 7.8 Hz, 2H, NCH-*H*), 1.35 (d, *J* = 6.1 Hz, 6H, C*H*_3_). ^13^C-NMR (101 MHz, CDCl_3_) δ 156.07 (N-*C*H=N), 137.16 (N-*C*H=CH), 129.43, 122.86, 121.57, 115.68 (Ph-*C*), 72.17 (O-*C*H), 53.83 (*C*H_2_), 16.42 (*C*H_3_). HRMS: *m/z* 337.1920 (calcd. 337.1916 for C_21_H_25_N_2_O_2_).

*1,3-Bis[2-(4-nitrophenoxy)propyl]imidazolium Bromide* (**7d**). Total yield 23.2%. Yellow oil. 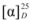 = −10.99 (*c* 0.56, CHCl_3_). IR: υ_C=N_ 1602 cm^−1^, υ_NO2_ 1508 cm^−1^, υ_C-O_ 1260 cm^−1^. ^1^H-NMR (400 MHz, CD_3_OD) δ 9.58 (s, 1H, N-C*H*=N), 8.07 (d, *J* = 2.1 Hz, 2H, N-C*H*), 8.05 (d, *J* = 2.0 Hz, 2H, Ph-*H*), 7.98 (d, *J* = 1.5 Hz, 2H, Ph-*H*), 7.16 (d, *J* = 2.1 Hz, 2H, Ph-*H*), 7.14 (d, *J* = 2.0 Hz, 2H, Ph-*H*), 5.23–5.15 (m, 2H, O-C*H*), 4.91 (dd, *J* = 14.3, 2.6 Hz, 2H, NCH-*H*), 4.74 (dd, *J* = 14.4, 8.4 Hz, 2H, NCH-*H*), 1.55 (d, *J* = 6.1 Hz, 6H, C*H*_3_). ^13^C-NMR (101 MHz, CD_3_OD) δ 163.13 (N-*C*H=N), 142.48 (Ph-*C*), 138.54 (N-*C*H=CH), 126.69, 124.49, 116.50 (Ph-*C*), 74.37 (O-*C*H), 54.80 (*C*H_2_), 16.76 (C*H*_3_). HRMS: *m/z* 427.1630 (calcd. 427.1618 for C_21_H_23_N_4_O_6_).

*1,3-Bis[2-(4-methylphenoxy)propyl]imidazolium Bromide* (**7e**). Total yield 22.7%. Yellow oil. 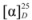 = −15.12 (*c* 1.025, CHCl_3_). IR: υ_C=N_ 1616 cm^−1^, υ_C-O_ 1092 cm^−1^. ^1^H-NMR (400 MHz, CDCl_3_) δ 10.04 (s, 1H, N-C*H*=N), 7.83 (d, *J* = 0.9 Hz, 2H, N-C*H*), 6.96 (d, *J* = 8.4 Hz, 4H, Ph-*H*), 6.71 (t, *J* = 5.9 Hz, 4H, Ph-*H*), 4.91 (dd, *J* = 13.8, 2.0 Hz, 2H, NCH-*H*), 4.77–4.70 (m, 2H, O-C*H*), 4.47 (dd, *J* = 13.9, 7.9 Hz, 2H, NCH-*H*), 2.22 (s, 6H, Ph-C*H*_3_), 1.33 (d, *J* = 6.2 Hz, 6H, CH-C*H*_3_). ^13^C-NMR (101 MHz, CDCl_3_) δ 153.54 (N-*C*H=N), 136.59 (N-*C*H=CH), 130.31, 129.33, 122.40, 115.28 (Ph-*C*), 72.02 (O-*C*H), 53.31 (*C*H_2_), 19.68 (CH-C*H*_3_), 15.95 (Ph-C*H*_3_). HRMS: *m/z* 365.2228 (calcd. 365.2229 for C_23_H_29_N_2_O_2_).

### 3.3. General Procedure for the Suzuki-Miyaura Coupling Reactions

Under an Ar atmosphere, Pd(OAc)_2_ (0.6 mg, 0.0025 mmol, 0.5 mol%), imidazolium salt (0.005 mmol, 1 mol%), arylboronic acid (0.75 mmol), aryl halide (0.5 mmol), K_2_CO_3_ (207.30 mg, 1.5 mmol), and toluene (3.0 mL) were added to a dried Schlenk tube in sequence. The mixture was stirred at 110 °C and the progress of the reaction was monitored by TLC and gas chromatography. Upon the consumption of aryl halide, the mixture was cooled to room temperature, and H_2_O (3.0 mL) was added to quench the reaction. The organic layer was separated, and the aqueous layer was back-extracted with CH_2_Cl_2_ (3.0 mL × 3). The organic phases were combined, dried over Na_2_SO_4_ and concentrated. The product was isolated by column chromatography with petroleum ether-ethyl acetate as the eluents or analyzed by gas chromatography using diethylene glycol di-*n*-butyl ether as an internal standard. The structures of the coupling products were confirmed by comparison of ^1^H-NMR, ^13^C-NMR with those reported in literature. All products, **12a**–**q**, showed molecular ionic peak in MS specta.

*Biphenyl* (**12a**): colorless solid, m.p. 69–70 °C (lit. [46], m.p. 69–70 °C).^1^H-NMR (400 MHz, CDCl_3_) δ 7.62–7.56 (m, 2H), 7.48–7.40 (m, 2H), 7.40–7.29 (m, 1H) ppm. ^13^C-NMR (101 MHz, CDCl_3_) δ 141.37, 128.89, 127.38, 127.30 ppm. The NMR data were in agreement with those reported in literature [46].

*4-Acetylbiphenyl* (**12b**): colorless solid, m.p. 122–124 °C (lit. [46], m.p. 120–121 °C). ^1^H-NMR (400 MHz, CDCl_3_) δ 8.04 (d, *J* = 8.3 Hz, 2H), 7.69 (d, *J* = 8.3 Hz, 2H), 7.65–7.60 (m, 2H), 7.48 (t, *J* = 7.4 Hz, 2H), 7.41 (d, *J* = 7.2 Hz, 1H), 2.64 (s, 3H) ppm. ^13^C-NMR (101 MHz, CDCl_3_) δ 197.62, 145.69, 139.82, 135.87, 128.96, 128.91, 128.24, 127.24, 127.17, 26.60 ppm. The NMR spectral data matched literature data [46,47].

*4-Nitrobiphenyl* (**12c**): yellow solid, m.p. 112–113 °C (lit. [46], m.p. 112–114 °C). ^1^H-NMR (400 MHz, CDCl_3_) δ 8.29 (d, *J* = 8.7 Hz, 2H), 7.73 (d, *J* = 8.7 Hz, 2H), 7.63 (d, *J* = 7.3 Hz, 2H), 7.49 (dt, *J* = 13.6, 7.0 Hz, 3H) ppm. ^13^C-NMR (101 MHz, CDCl_3_) δ 147.62, 147.06, 138.74, 129.21, 128.99, 127.82, 127.42, 124.14 ppm. The NMR spectral data matched literature data [46,47].

*4-Cyanobiphenyl* (**12d**): colorless solid, m.p. 86–87 °C (lit. [46], m.p. 85–86 °C). ^1^H-NMR (400 MHz, CDCl_3_) δ 7.73–7.60 (m, 4H), 7.59–7.52 (m, 2H), 7.50–7.33 (m, 3H) ppm. ^13^C-NMR (101 MHz, CDCl_3_) δ 145.55, 139.05, 132.55, 129.10, 128.66, 127.67, 127.18, 118.95, 110.81 ppm. The NMR spectral data matched literature data [46,47].

*Methyl biphenyl-4-carboxylate* (**12e**): colorless solid, m.p. 119–120 °C (lit. [46], m.p. 115–116 °C). ^1^H-NMR (400 MHz, CDCl_3_) δ 8.13 (d, *J* = 8.3 Hz, 2H), 7.65 (dd, *J* = 14.4, 7.8 Hz, 4H), 7.44 (dt, *J* = 26.9, 7.2 Hz, 3H), 3.95 (s, 3H) ppm. ^13^C-NMR (101 MHz, CDCl_3_) δ 166.96, 145.58, 139.94, 130.12, 128.94, 128.87, 128.16, 127.27, 127.03, 52.13 ppm. The NMR data are in agreement to those in literature [46,47].

*N-Acetyl-4-aminobiphenyl* (**12f**): colorless solid, m.p. 172–174 °C (lit. [46], m.p. 171–172 °C). ^1^H-NMR (400 MHz, CD_3_OD) δ 7.65–7.50 (m, 6H), 7.39 (t, *J* = 7.6 Hz, 2H), 7.28 (t, *J* = 7.3 Hz, 1H), 2.13 (s, 3H) ppm. ^13^C-NMR (101 MHz, CD_3_OD) δ 171.60, 141.81, 139.21, 138.13, 129.80, 128.20, 128.04, 127.64, 121.41, 23.84 ppm. The NMR spectral data matched literature data [46,48].

*4-chlorobiphenyl* (**12g**): colorless solid, m.p. 77–78 °C (lit. [46], m.p. 78–79 °C, lit. [49], m.p. 75–78 °C). ^1^H-NMR (400 MHz, CDCl_3_) δ 7.56 (dd, *J* = 14.8, 7.9 Hz, 4H), 7.50–7.35 (m, 5H) ppm. ^13^C-NMR (101 MHz, CDCl_3_) δ 140.09, 139.76, 133.48, 129.03, 129.01, 128.51, 127.71, 127.11 ppm. The NMR spectral data matched literature data [46,49].

*4-Phenylphenol* (**12h**): colorless solid, m.p. 164–166 °C (lit. [50], m.p. 162–164 °C, lit. [51], m.p. 167.2–168.0 °C). ^1^H-NMR (400 MHz, CD_3_OD) δ 7.48 (dd, *J* = 8.2, 1.0 Hz, 2H), 7.45–7.37 (m, 2H), 7.32 (t, *J* = 7.7 Hz, 2H), 7.24–7.16 (m, 1H), 6.92–6.83 (m, 2H) ppm. ^13^C-NMR (101 MHz, CD_3_OD) δ 157.93, 142.18, 133.74, 129.60, 128.96, 127.30, 127.28, 116.53, 49.43, 49.21, 49.00, 48.79, 48.57 ppm. The NMR spectral data matched literature data [50,51]. MS (EI): *m/z* = 170 [M^+^].

*2-Butyoxyl-5-tert-butylbiphenyl* (**12i**): colorless oil. ^1^H-NMR (400 MHz, CDCl_3_) δ 7.70–7.65 (m, 2H), 7.53–7.46 (m, 3H), 7.44–7.38 (m, 2H), 7.02 (d, *J* = 8.6 Hz, 1H), 4.04 (t, *J* = 6.4 Hz, 2H), 1.79 (dt, *J* = 14.3, 6.5 Hz, 2H), 1.52 (dd, *J* = 14.9, 7.5 Hz, 2H), 1.45 (s, 9H), 1.01 (t, *J* = 7.4 Hz, 3H) ppm. ^13^C-NMR (101 MHz, CDCl_3_) δ 154.00, 143.45, 139.37, 130.46, 129.80, 128.24, 127.90, 126.74, 125.24, 112.30, 77.48, 77.16, 76.84, 68.35, 34.28, 31.71, 31.50, 19.45, 13.91 ppm. 

*4-Methylbiphenyl* (**12j**): colorless solid, m.p. 42–44 °C (lit. [52], m.p. 41–42 °C). ^1^H-NMR (400 MHz, CDCl_3_) δ 7.70–7.65 (m, 2H), 7.59 (d, *J* = 8.1 Hz, 2H), 7.51 (t, *J* = 7.6 Hz, 2H), 7.41 (dd, *J* = 11.6, 4.3 Hz, 1H), 7.33 (d, *J* = 7.9 Hz, 2H), 2.48 (s, 3H) ppm. ^13^C-NMR (101 MHz, CDCl_3_) δ 141.30, 138.50, 137.11, 129.60, 128.83, 127.12, 127.09, 21.22 ppm. The NMR spectral data matched literature data [52,53].

*2-Methylbiphenyl* (**12k**): colorless oil. ^1^H-NMR (400 MHz, CDCl_3_) δ 7.53–7.47 (m, 2H), 7.42 (d, *J* = 7.2 Hz, 3H), 7.39–7.30 (m, 4H), 2.37 (s, 3H) ppm. ^13^C-NMR (101 MHz, CDCl_3_) δ 142.06, 142.04, 135.44, 130.42, 129.91, 129.30, 128.18, 127.36, 126.87, 125.88, 20.61 ppm. The NMR spectral data matched literature data [52,54]. 

*2,5-Dimethylbiphenyl* (**12l**): colorless solid, m.p. 68–70 °C. ^1^H-NMR (400 MHz, CDCl_3_) δ 7.40–7.35 (m, 2H), 7.30 (d, *J* = 7.1 Hz, 3H), 7.15 (d, *J* = 7.9 Hz, 1H), 7.06 (d, *J* = 6.7 Hz, 2H), 2.33 (s, 3H), 2.22 (s, 3H) ppm. ^13^C-NMR (101 MHz, CDCl_3_) δ 142.21, 141.87, 135.28, 132.27, 130.66, 130.37, 129.29, 128.14, 128.06, 126.80, 21.06, 20.10 ppm. The NMR spectral data matched literature data [55,56].

*2,6-Dimethylbiphenyl* (**12m**): colorless oil. ^1^H-NMR (400 MHz, CDCl_3_) δ 7.37 (t, *J* = 7.5 Hz, 2H), 7.28 (t, *J* = 7.3 Hz, 1H), 7.15–7.03 (m, 5H), 1.99 (s, 6H) ppm. ^13^C-NMR (101 MHz, CDCl_3_) δ 142.01, 141.25, 136.17, 129.16, 128.54, 127.41, 127.15, 126.73, 20.97 ppm. The NMR spectral data matched literature data [52,57]. 

*3,5-Di-tert-butylbiphenyl* (**12n**): colorless solid, m.p. 63–64 °C (lit. [58], m.p. 62–63 °C). ^1^H-NMR (400 MHz, CDCl_3_) δ 7.69–7.64 (m, 2H), 7.55–7.45 (m, 5H), 7.40 (dd, *J* = 6.9, 1.1 Hz, 1H), 1.45 (d, *J* = 2.3 Hz, 18H) ppm. ^13^C-NMR (101 MHz, CDCl_3_) δ 151.25, 142.70, 140.87, 128.77, 127.62, 127.09, 121.87, 121.53, 35.14, 31.71 ppm. The NMR spectral data matched literature data [58]. 

*Diphenylmethane* (**12o**): colorless solid, m.p. 20–22 °C (lit. [59], m.p. 21–24 °C). ^1^H-NMR (400 MHz, CDCl_3_) δ 7.27 (d, *J* = 7.2 Hz, 4H), 7.18 (d, *J* = 6.5 Hz, 6H), 3.98 (s, 2H) ppm. ^13^C-NMR (101 MHz, CDCl_3_) δ 141.24, 129.06, 128.58, 126.19, 42.07 ppm. The NMR spectral data matched literature data [59,60].

*3-Methylbiphenyl* (**12p**): colorless oil. ^1^H-NMR (400 MHz, CDCl_3_) δ 7.48 (d, *J* = 7.2 Hz, 2H), 7.31 (t, *J* = 10.4 Hz, 4H), 7.22 (t, *J* = 7.1 Hz, 2H), 7.06 (d, *J* = 7.1 Hz, 1H), 2.31 (s, 3H) ppm. ^13^C-NMR (101 MHz, CDCl_3_) δ 141.46, 141.34, 138.42, 128.81, 128.79, 128.12, 128.09, 127.29, 124.39, 21.68 ppm. The NMR spectral data matched literature data [61].

*Methyl 1-(4′-acetyl-biphenyl-4-yl)-carboxylate* (**12q**): colorless solid, m.p. 165–168 °C (lit. [62], m.p. 164.5–166 °C). ^1^H-NMR (400 MHz, CDCl_3_) δ 8.14 (d, *J* = 8.4 Hz, 2H), 8.05 (d, *J* = 8.4 Hz, 2H), 7.71 (t, *J* = 8.8 Hz, 4H), 3.95 (s, 3H), 2.65 (s, 3H) ppm. ^13^C-NMR (101 MHz, CDCl_3_) δ 197.68, 166.87, 144.57, 144.32, 136.65, 130.34, 129.91, 129.10, 127.56, 127.35, 52.34, 26.80 ppm. The NMR spectral data matched literature data [62,63].

## 4. Conclusions

In conclusion, we have synthesized a range of novel *C_2_*-symmetric NHC precursor imidazolium salts containing side arms substituted with aryloxyl groups. The Suzuki-Miyaura coupling reaction could be catalyzed remarkably by the Pd/NHC catalysts formed *in situ*. Various functionalized and sterically hindered aryl halides and arylboronic acids could be used. TON of up to 10^5^ was achieved with 5 × 10^−4^ mol% Pd catalyst.

## Supplementary Materials

Supplementary materials can be accessed at: http://www.mdpi.com/1420-3049/17/10/12121/s1.
