# Supplementary material for: Suzuki-Miyaura Reactions Catalyzed by C2-Symmetric Pd-Multi-Dentate N-Heterocyclic Carbene Complexes
Source: Molecules. 2012 Oct 16;17(10):12121–39. doi: 10.3390/molecules171012121 (PMC6268461; doi:10.3390/molecules171012121)

1. ^{1}H-NMR and ^{13}C-NMR spectra of the Suzuki-Miyaura Cross-coupling products 12a–q

Figure S1. ^{1}H-NMR spectrum of **12a**.

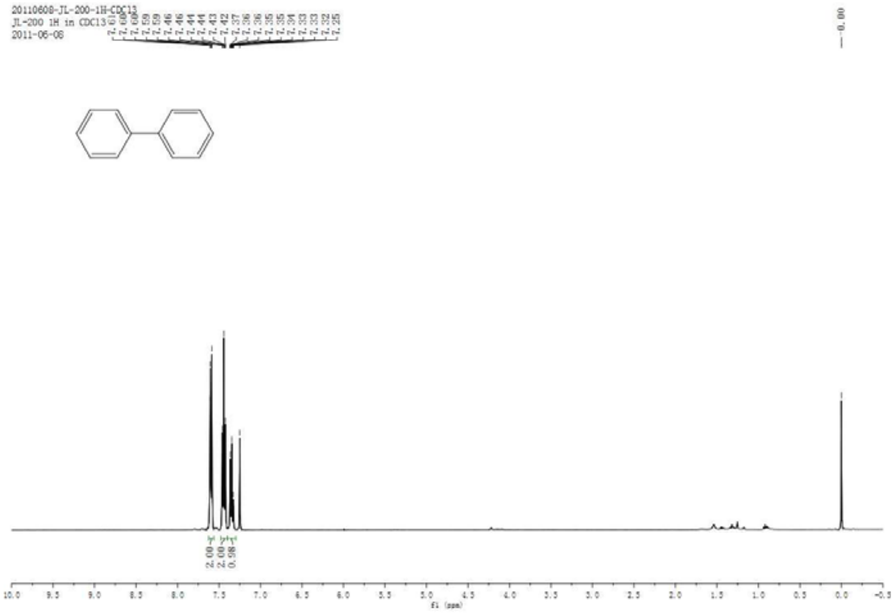

Figure S2. ^{13}C-NMR spectrum of **12a**.

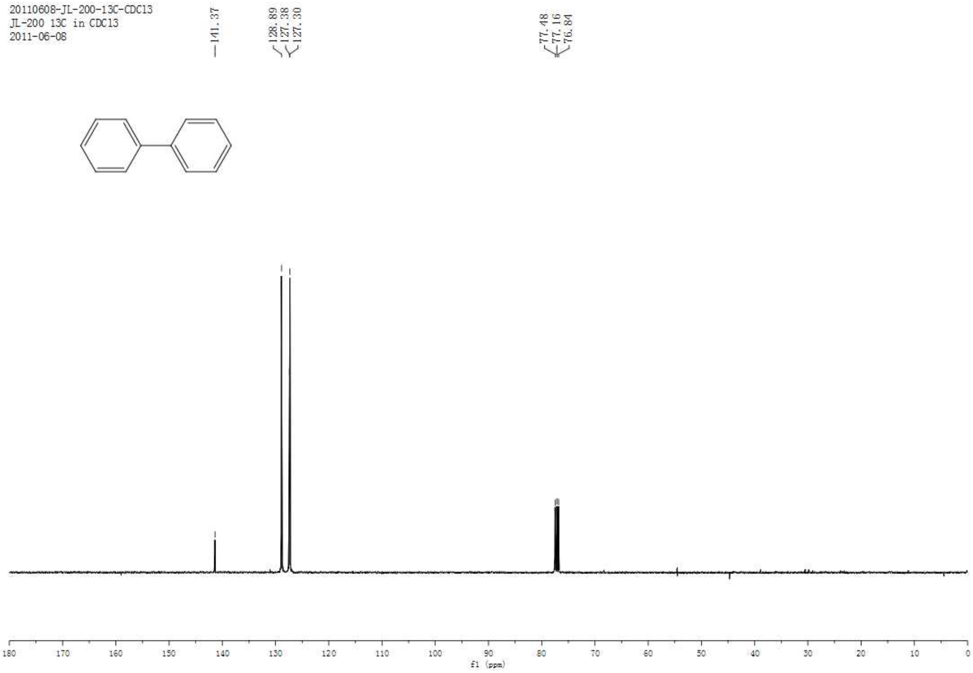

Figure S3. ^{1}H-NMR spectrum of **12b**.

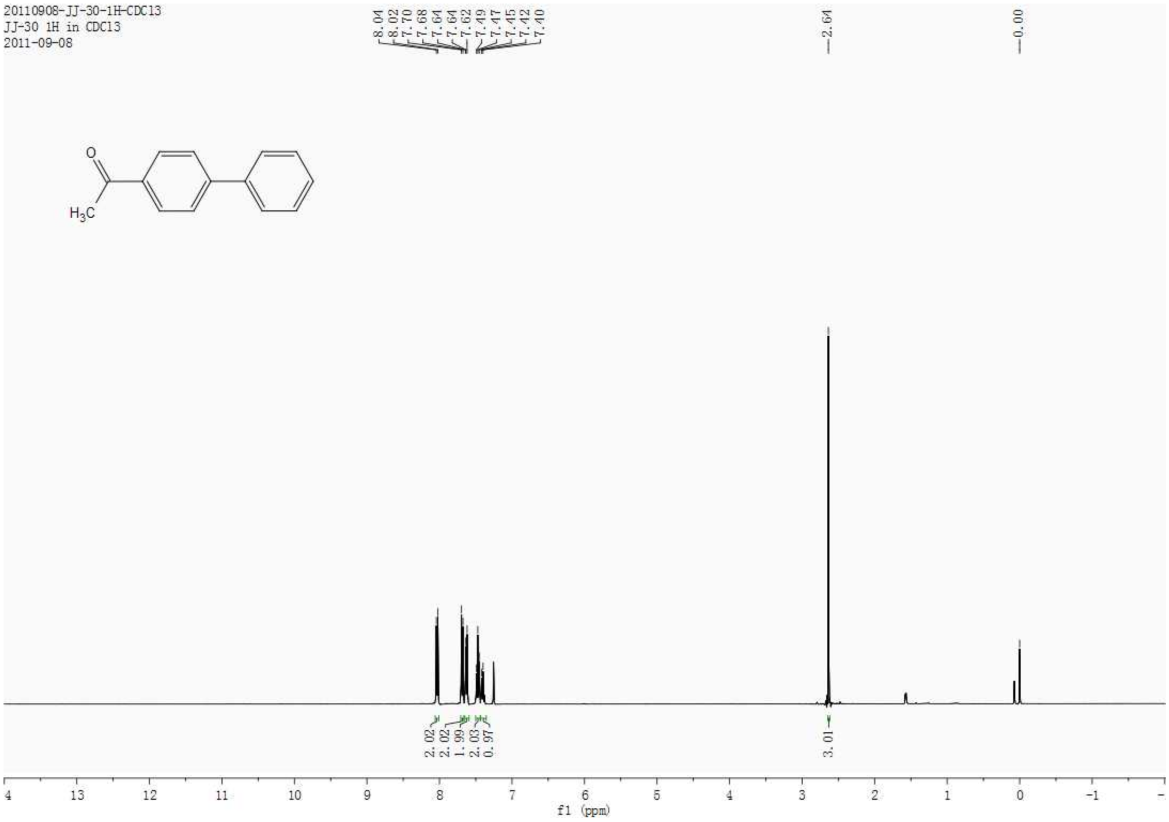

Figure S4. ^{13}C-NMR spectrum of **12b**.

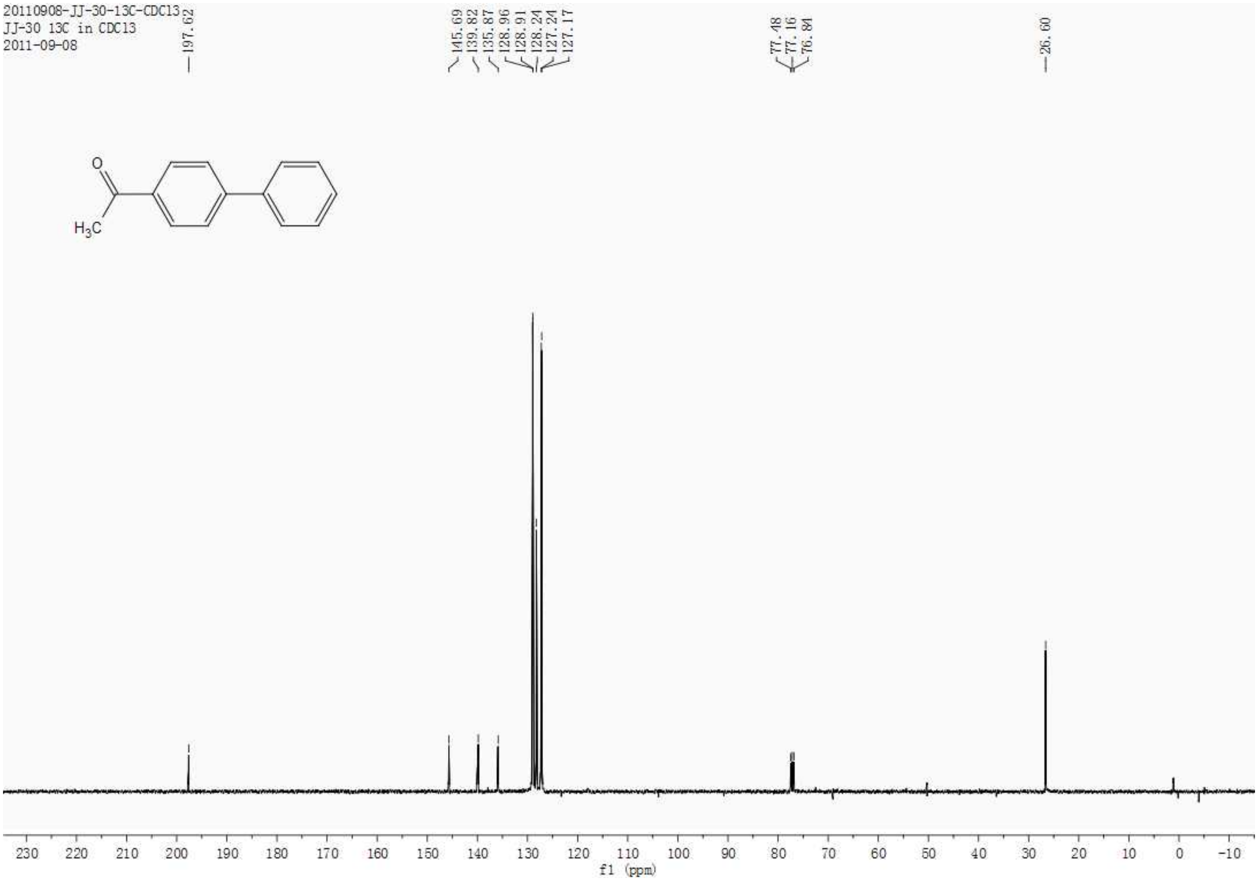

Figure S5. ^{1}H-NMR spectrum of **12c**.

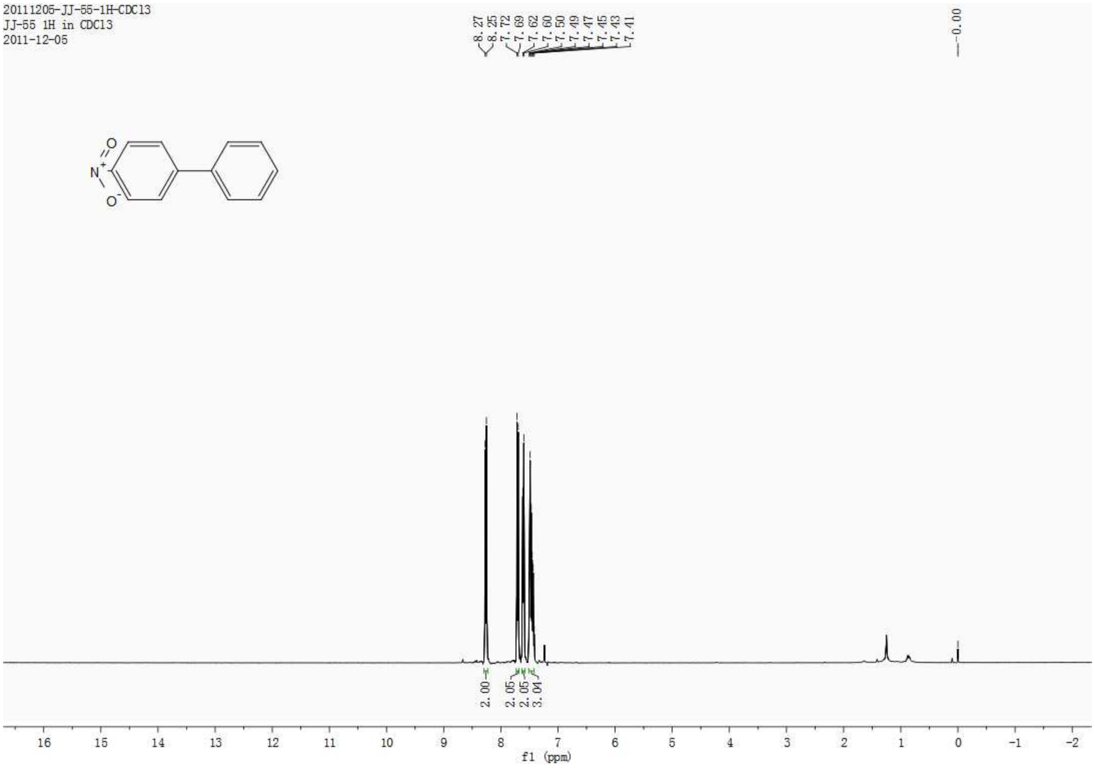

Figure S6. ^{13}C-NMR spectrum of **12c**.

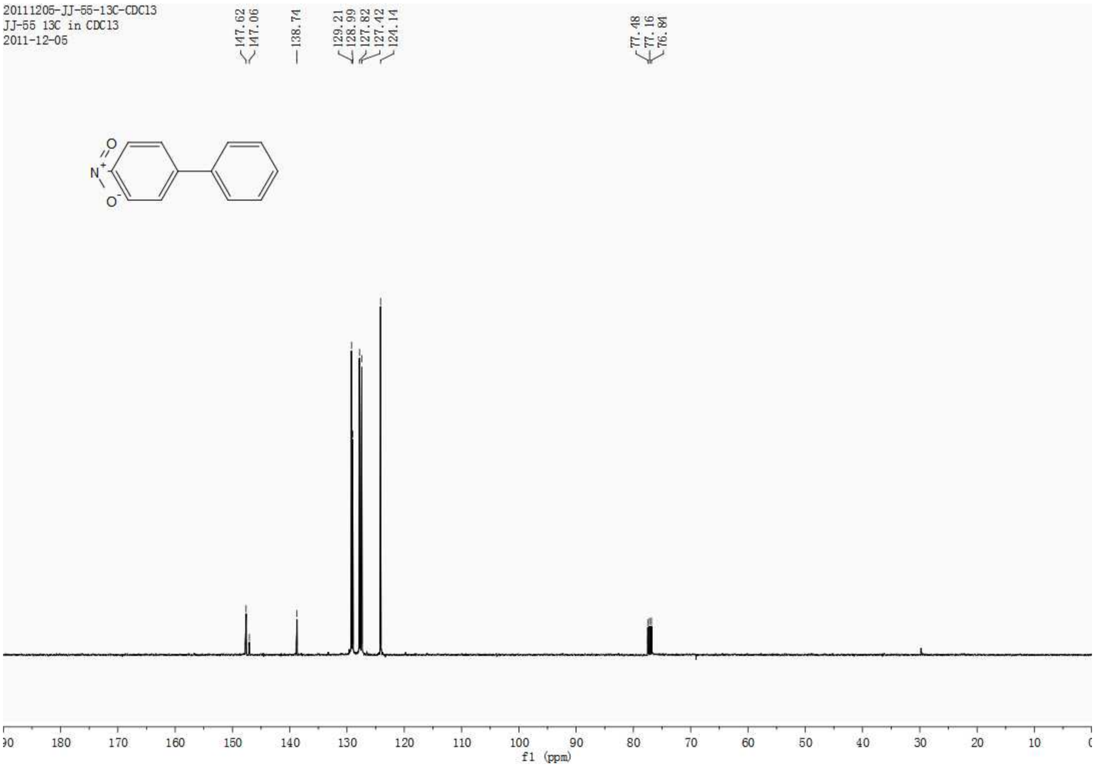

Figure S7. 1H-NMR spectrum of **12d**.

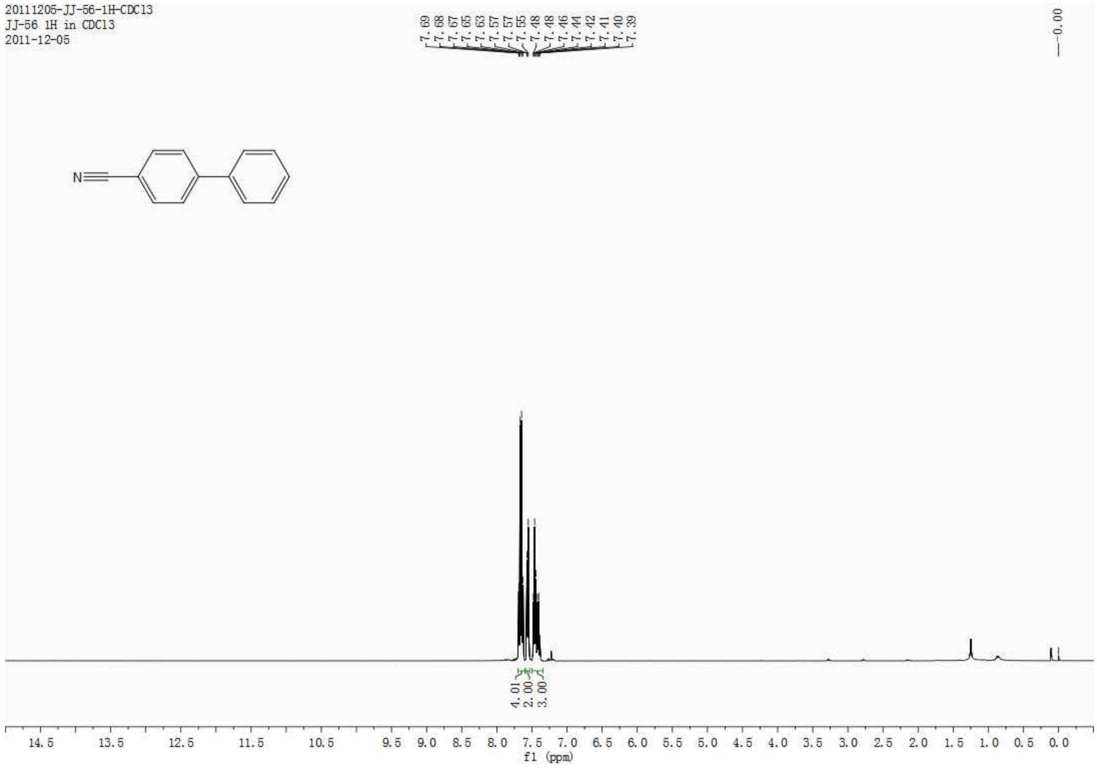

Figure S8. ^{13}C-NMR spectrum of **12d**.

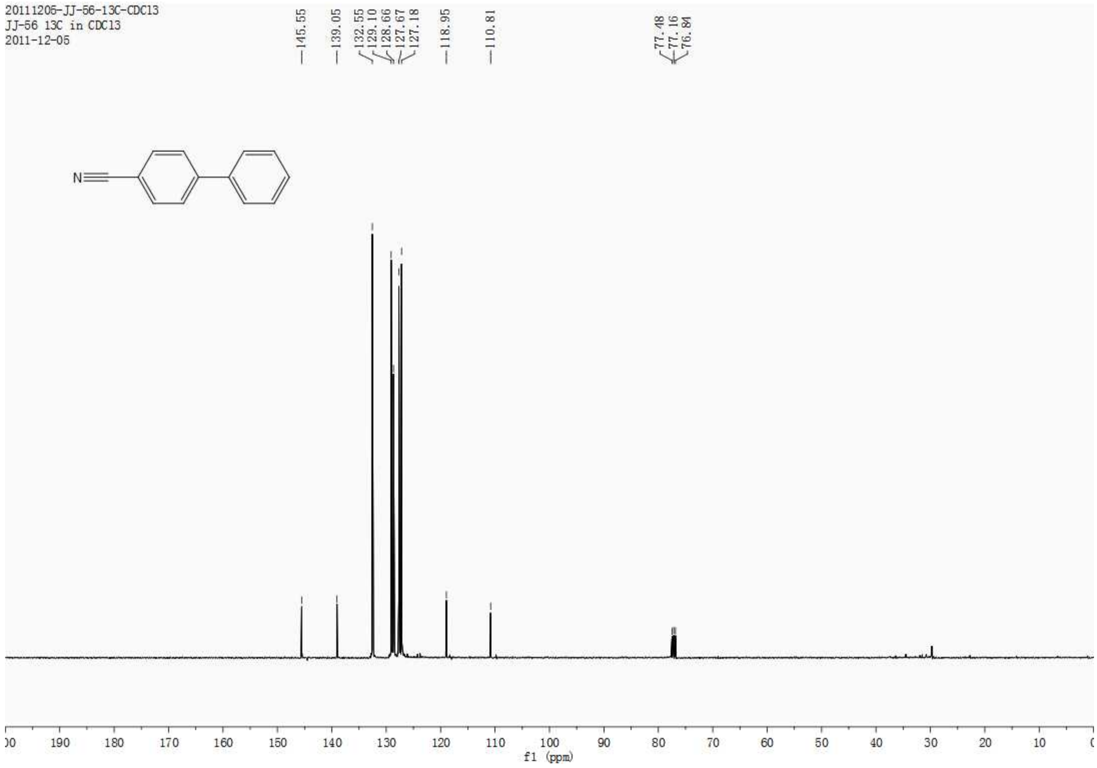

Figure S9. ^{1}H-NMR spectrum of **12e**.

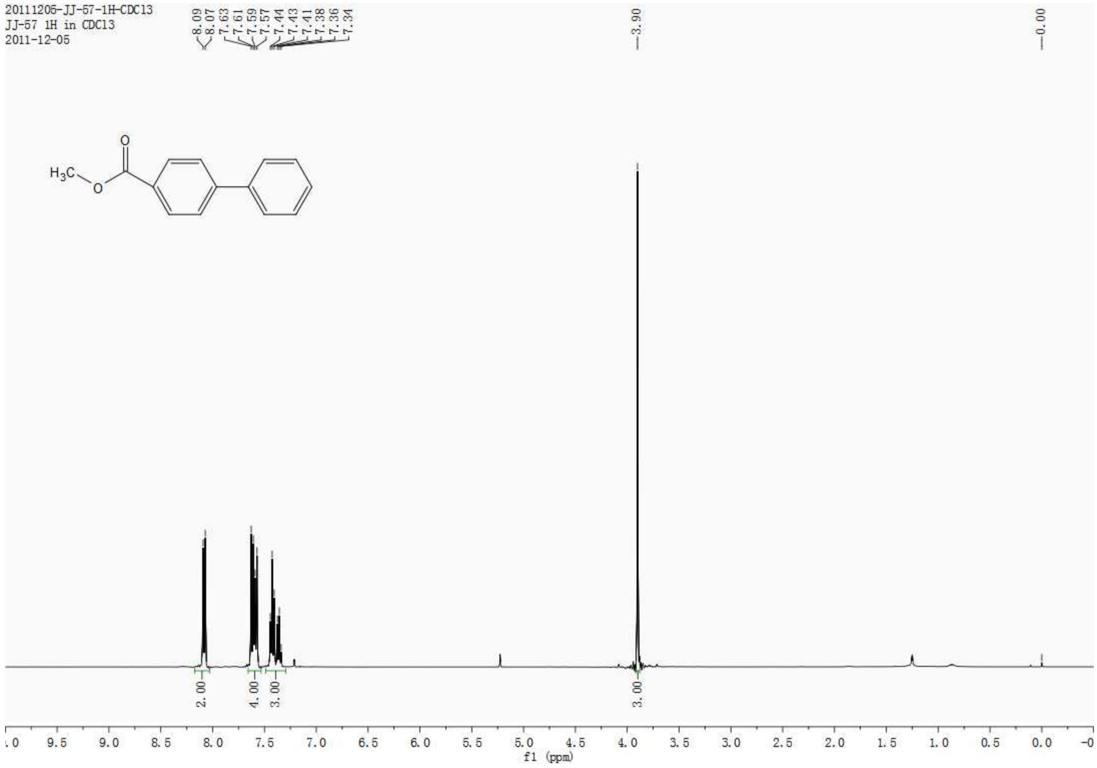

Figure S10. ^{13}C-NMR spectrum of **12e**.

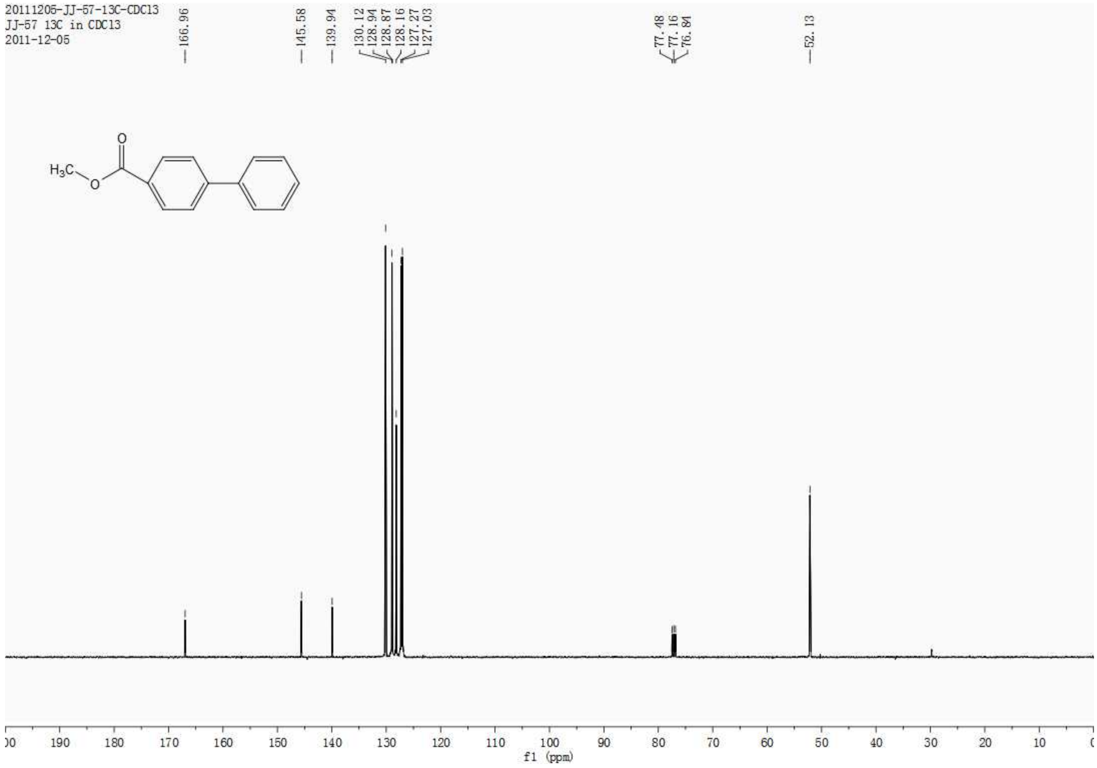

Figure S11. ^{1}H-NMR spectrum of **12f**.

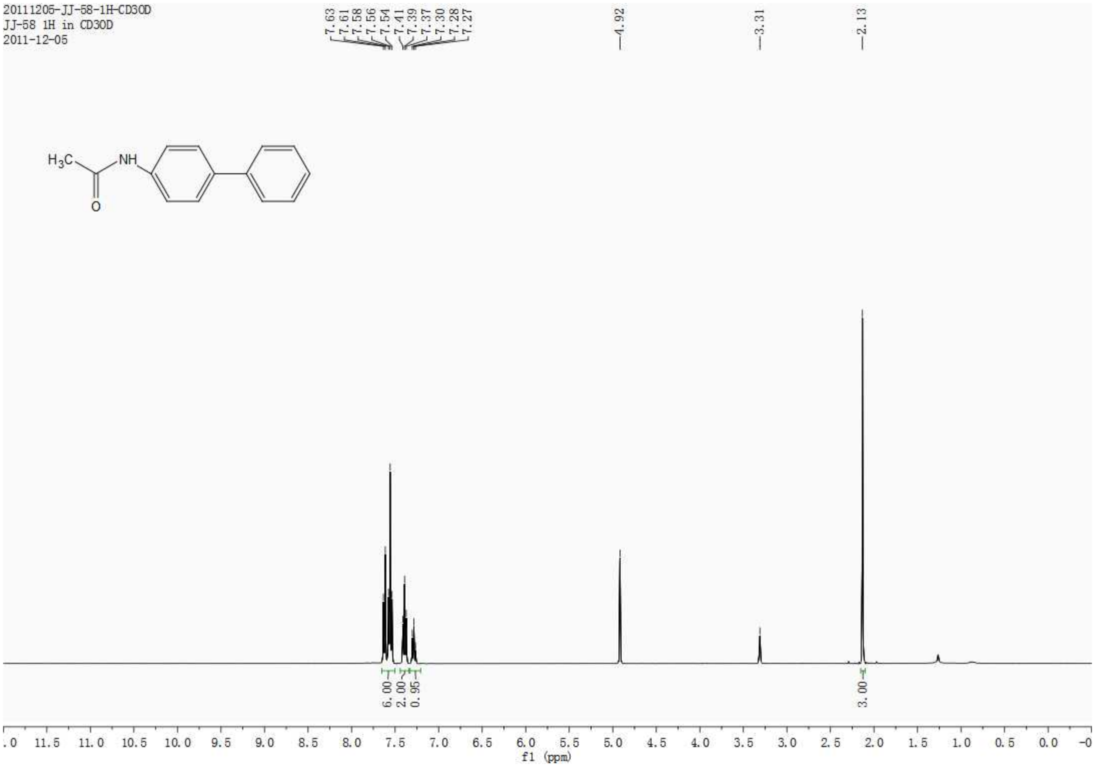

Figure S12. ^{13}C-NMR spectrum of **12f**.

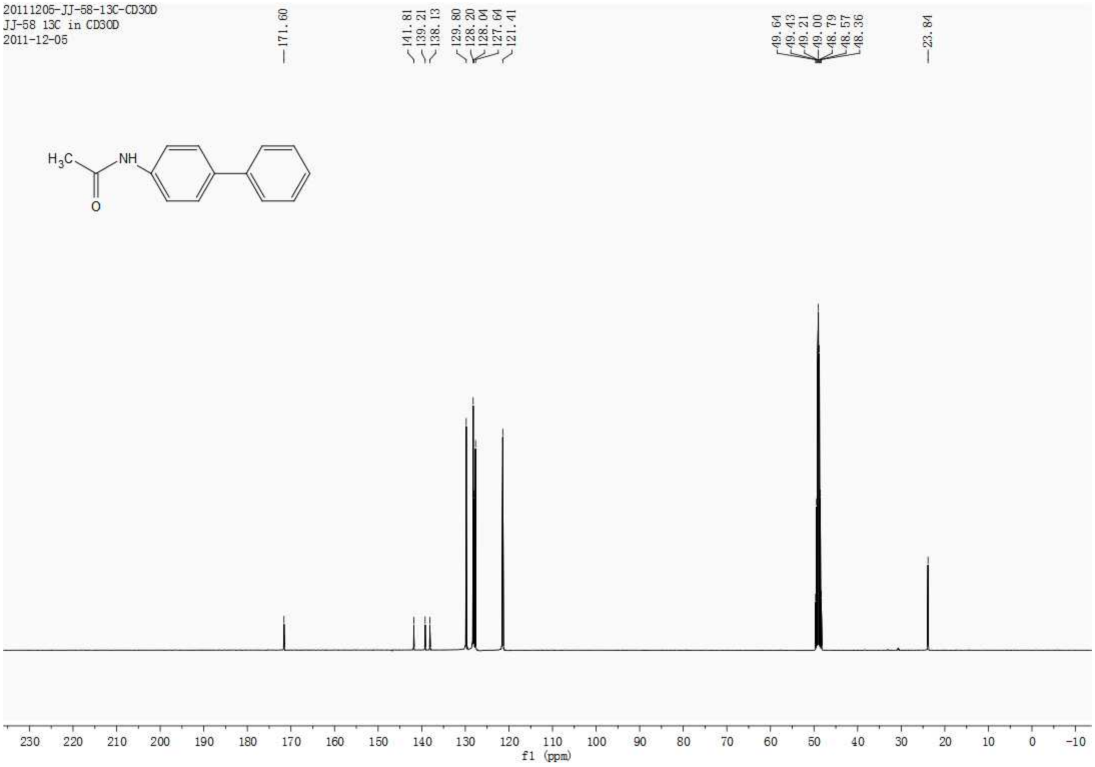

Figure S13. ^{1}H-NMR spectrum of **12g**.

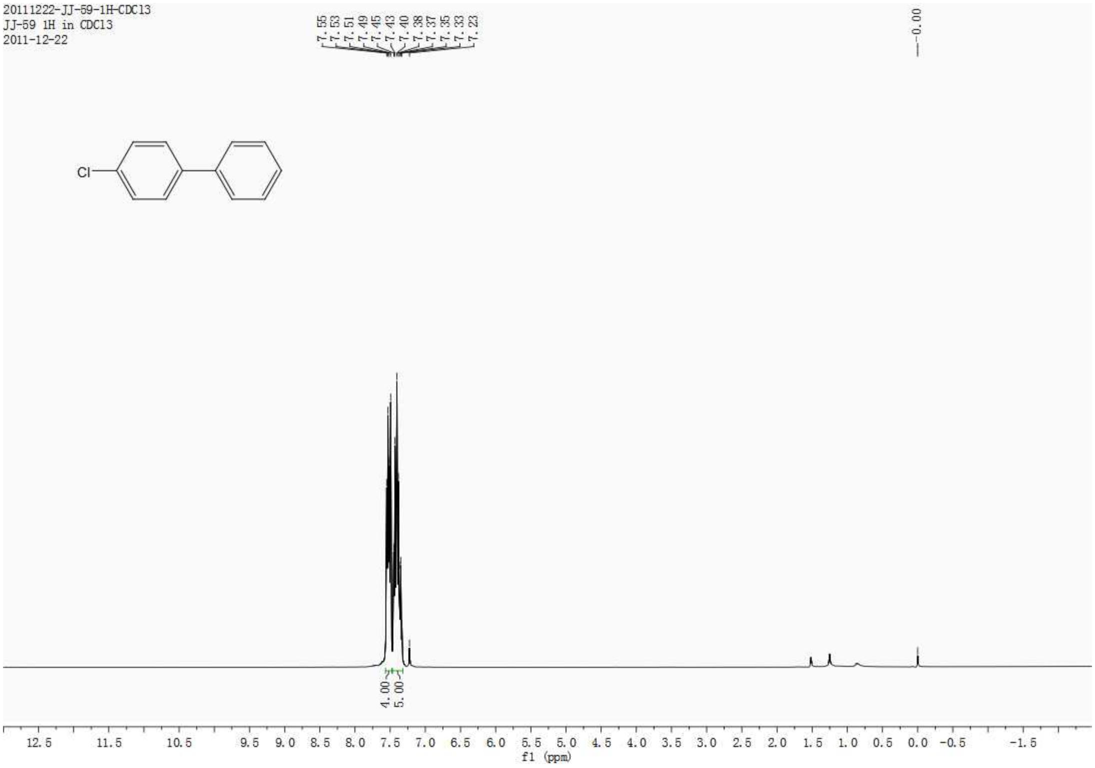

Figure S14. ^{13}C-NMR spectrum of **12g**.

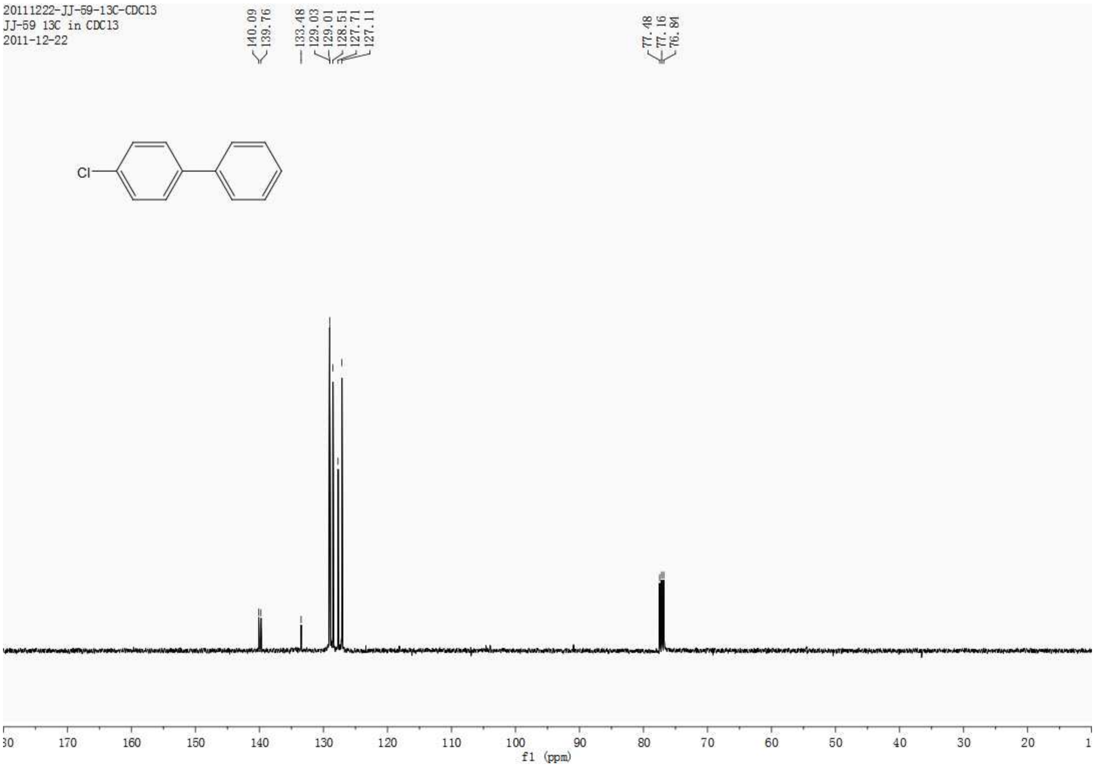

Figure S15. ^{1}H-NMR spectrum of **12h**.

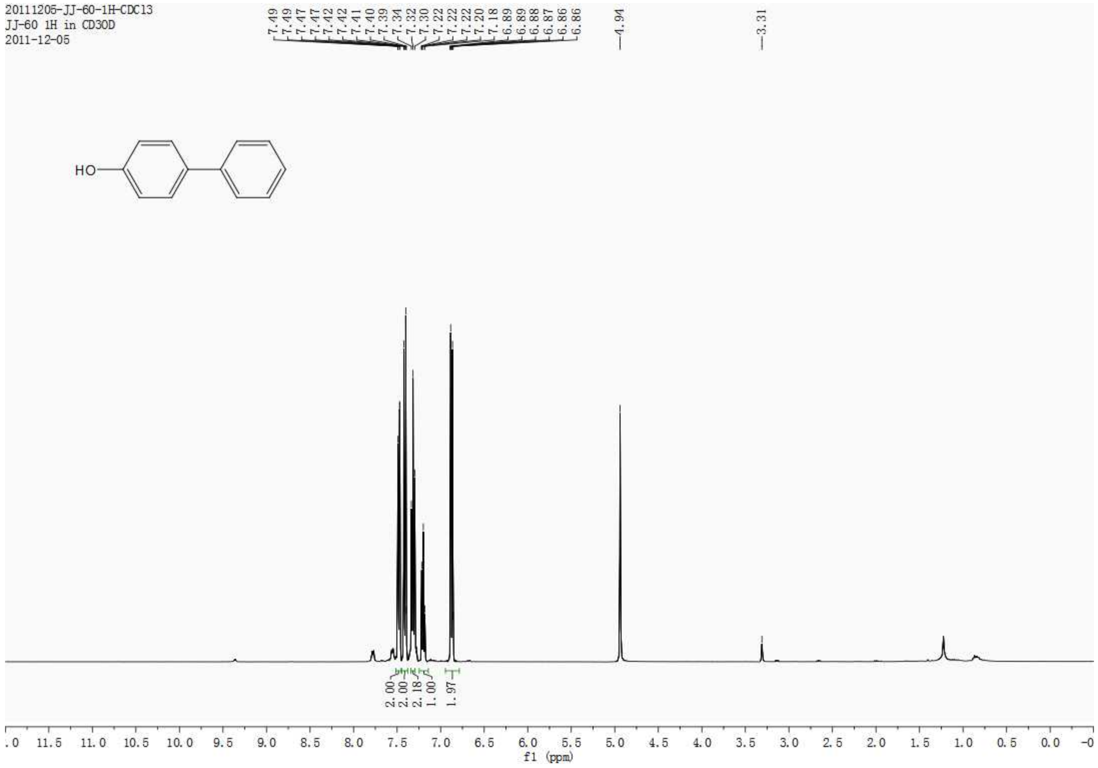

Figure S16. ^{13}C-NMR spectrum of **12h**.

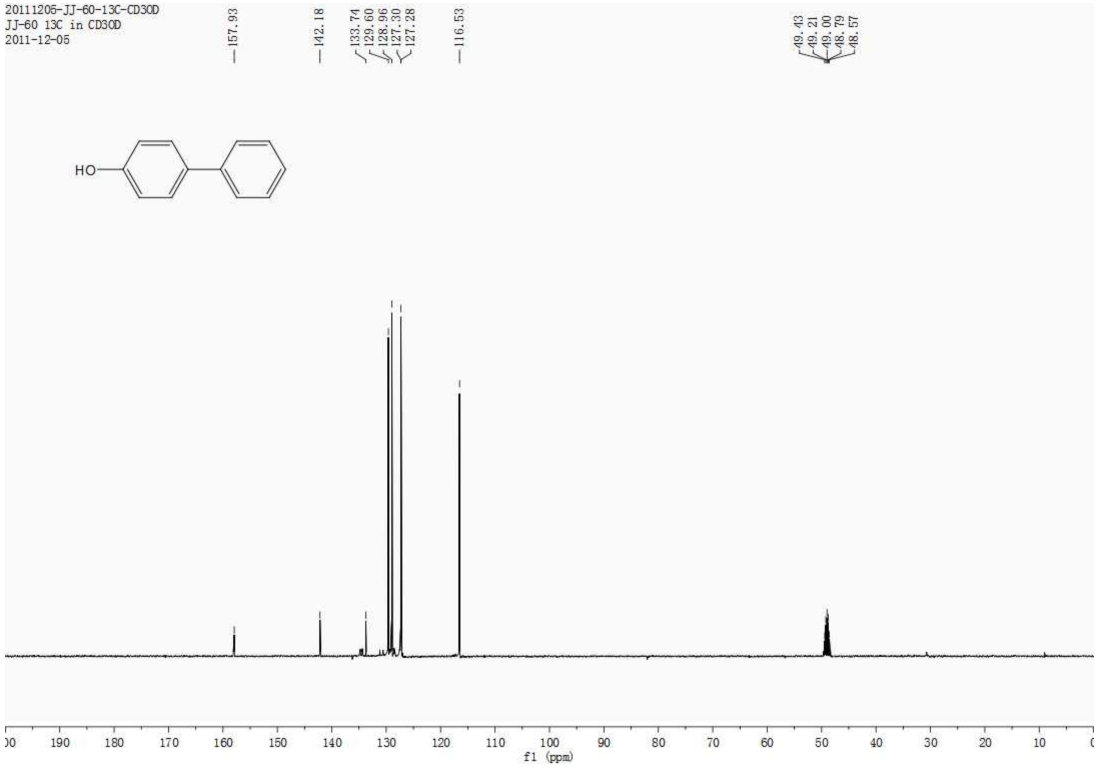

Figure S17. ^{1}H-NMR spectrum of **12i**.

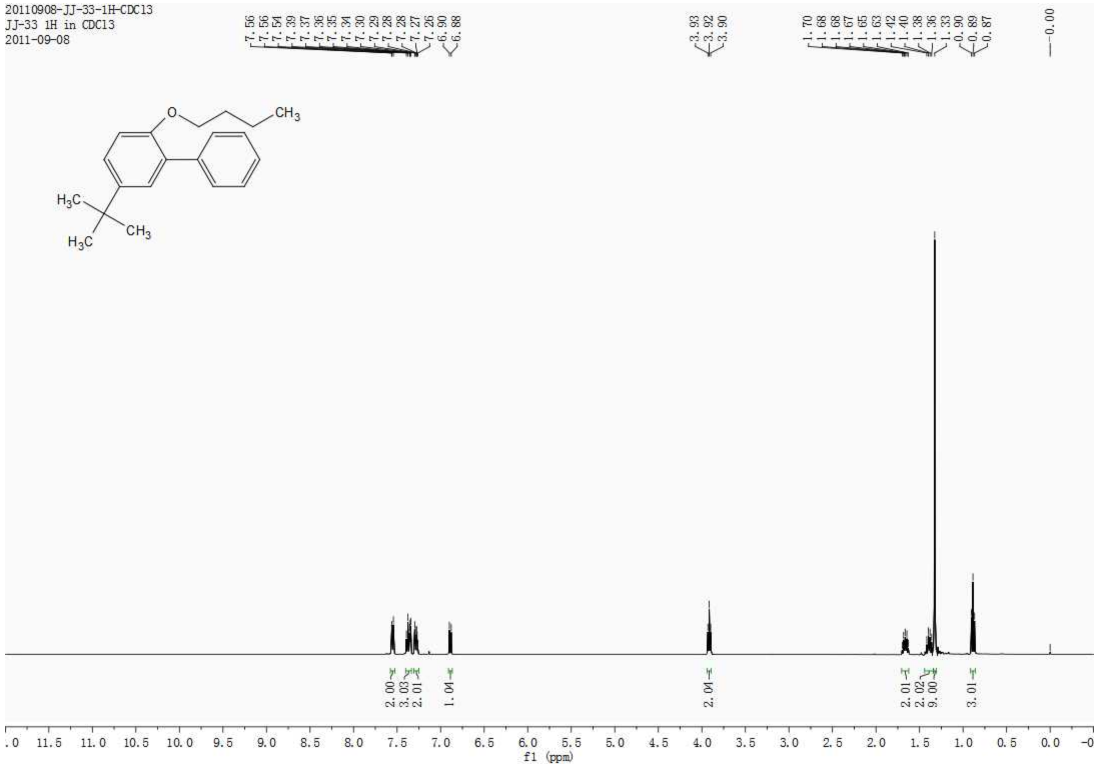

Figure S18. ^{13}C-NMR spectrum of **12i**.

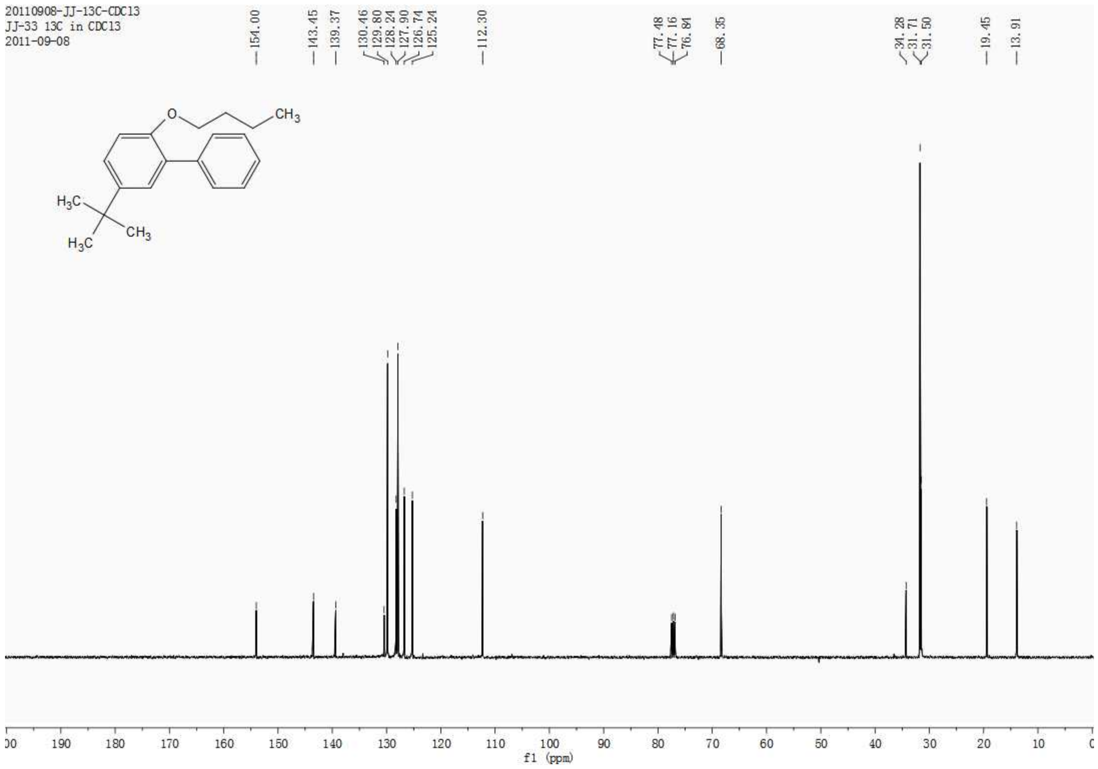

Figure S19. ^{1}H-NMR spectrum of **12j**.

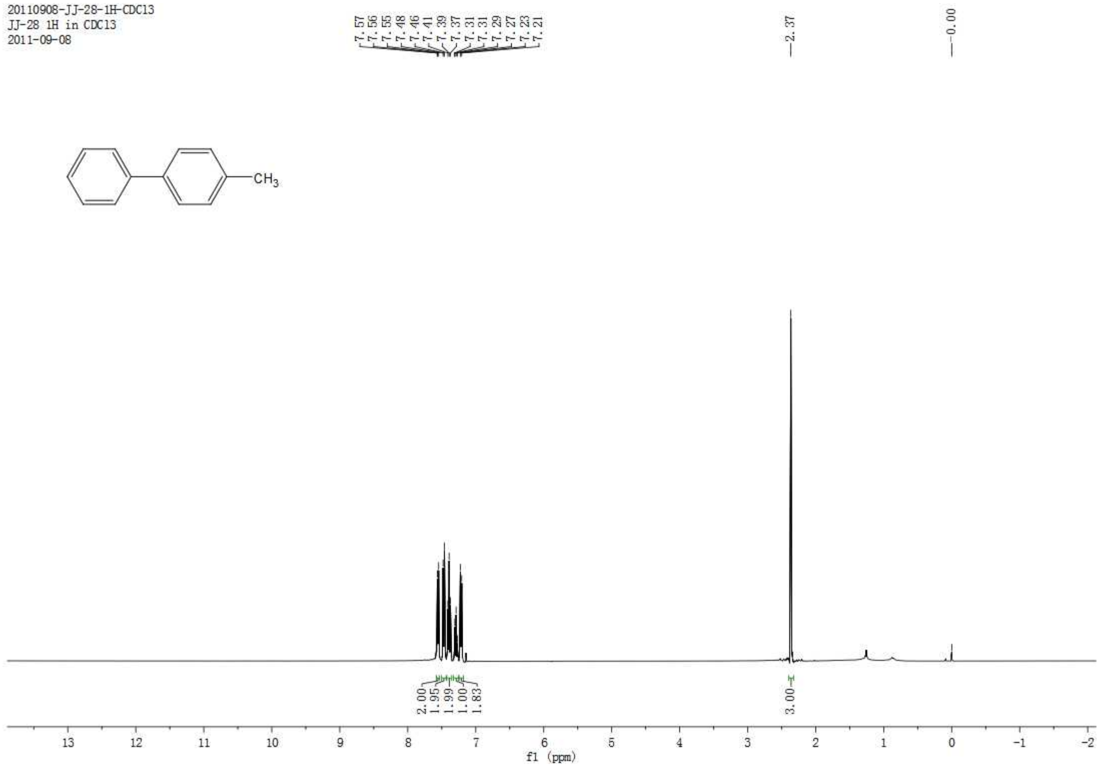

Figure S20. ^{13}C-NMR spectrum of **12j**.

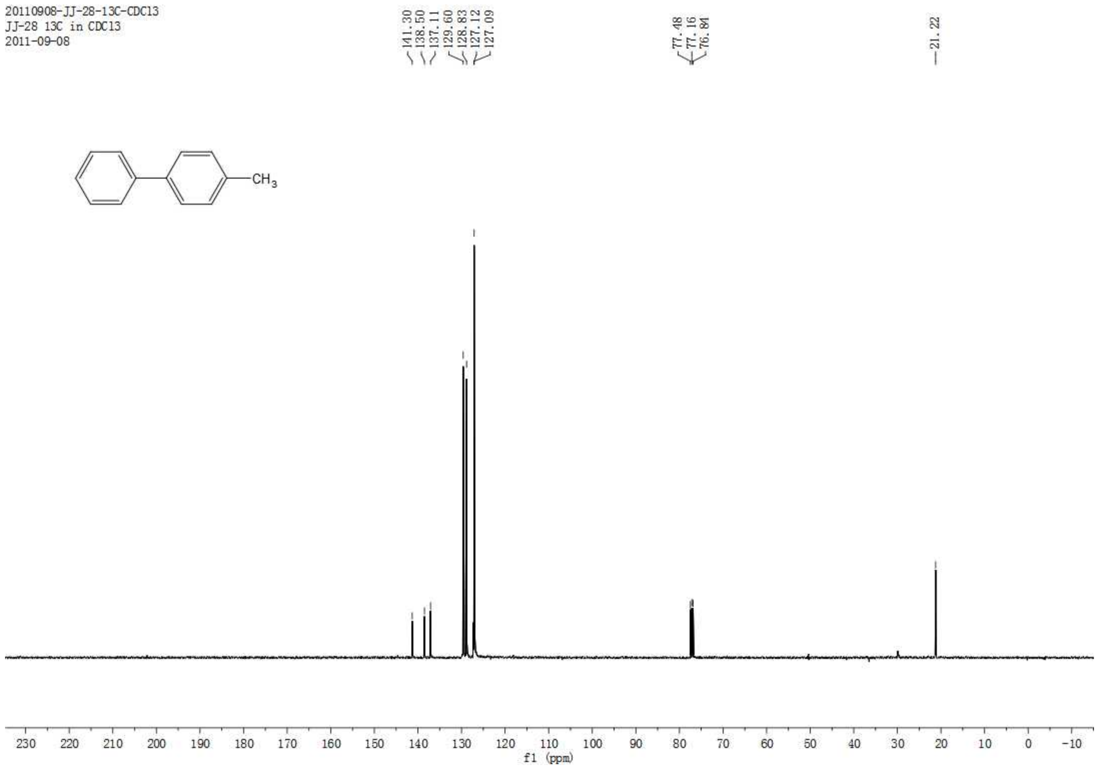

Figure S21. ^{1}H-NMR spectrum of **12k**.

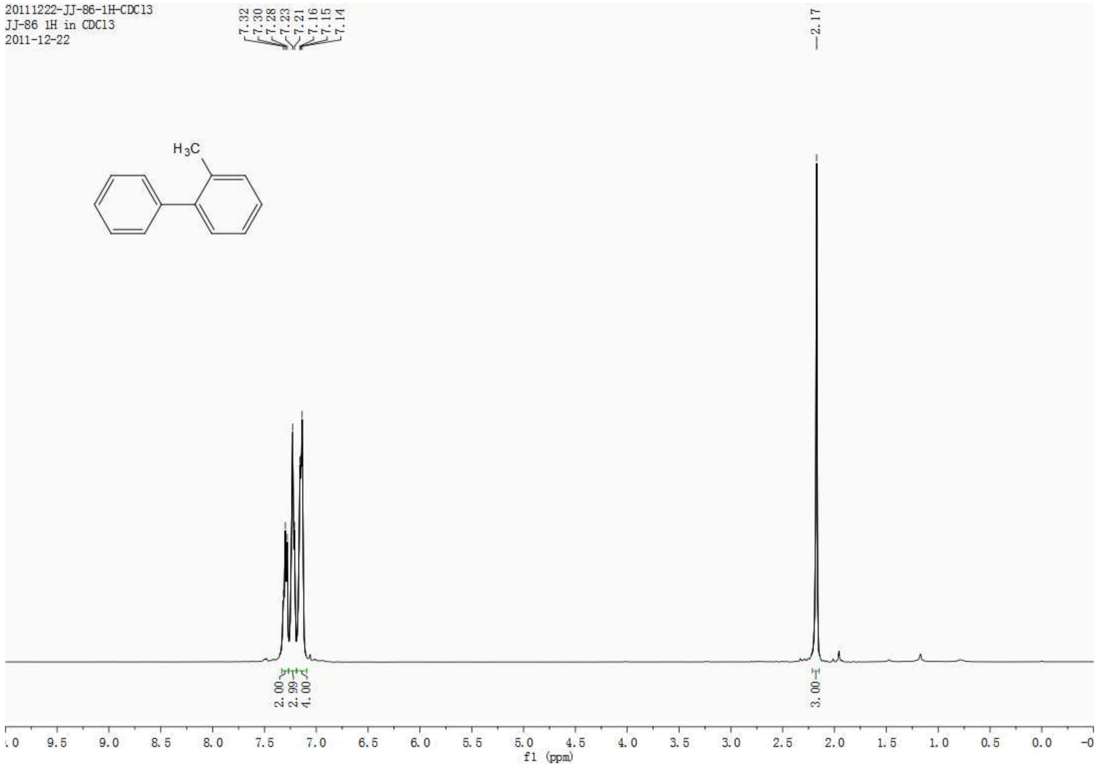

Figure S22. ^{13}C-NMR spectrum of **12k**.

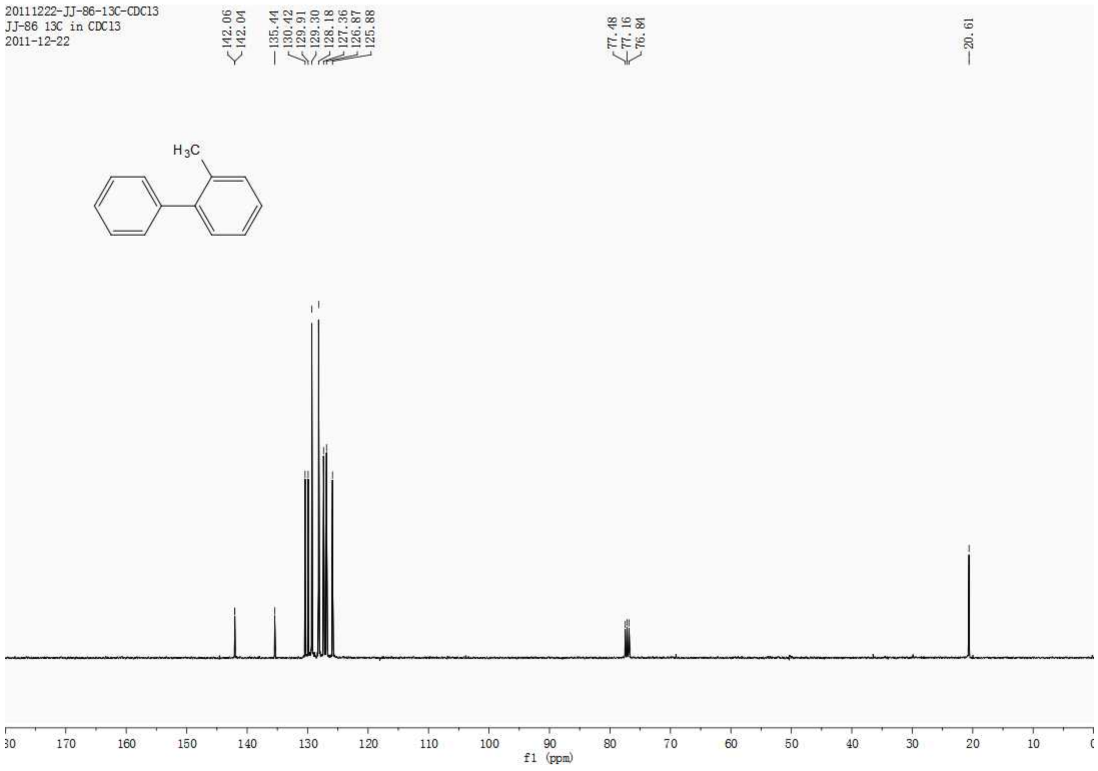

Figure S23. ^{1}H-NMR spectrum of **12l**.

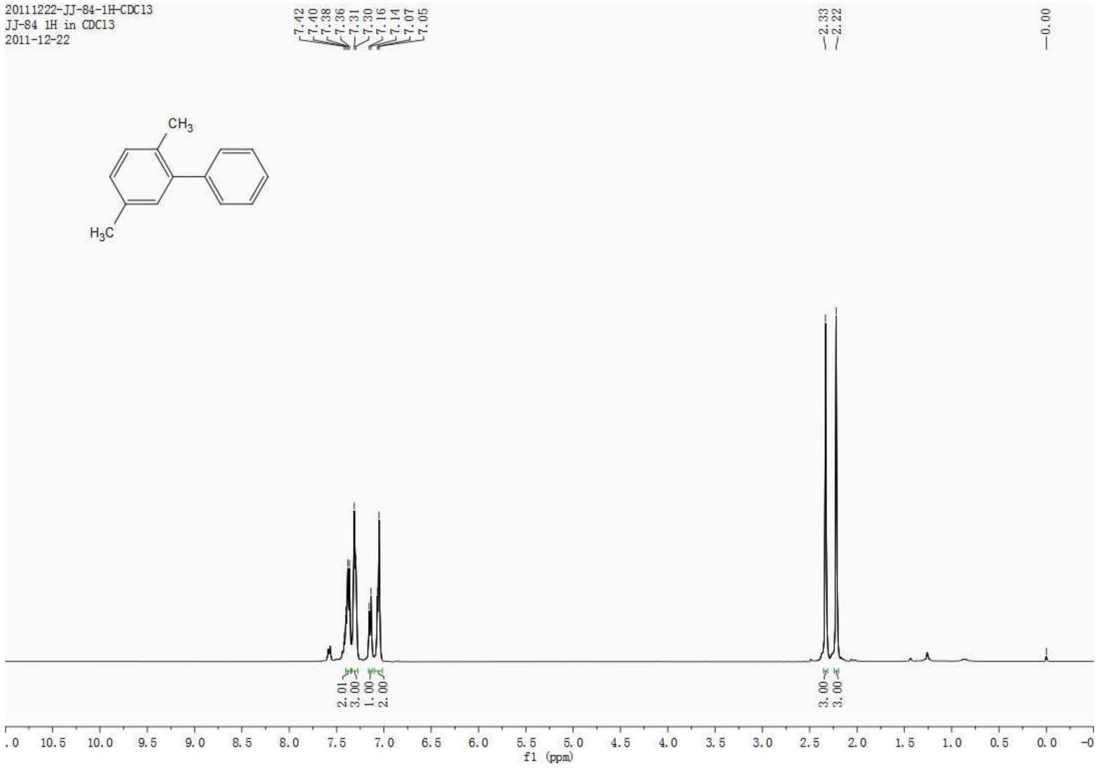

Figure S24. ^{13}C-NMR spectrum of **12l**.

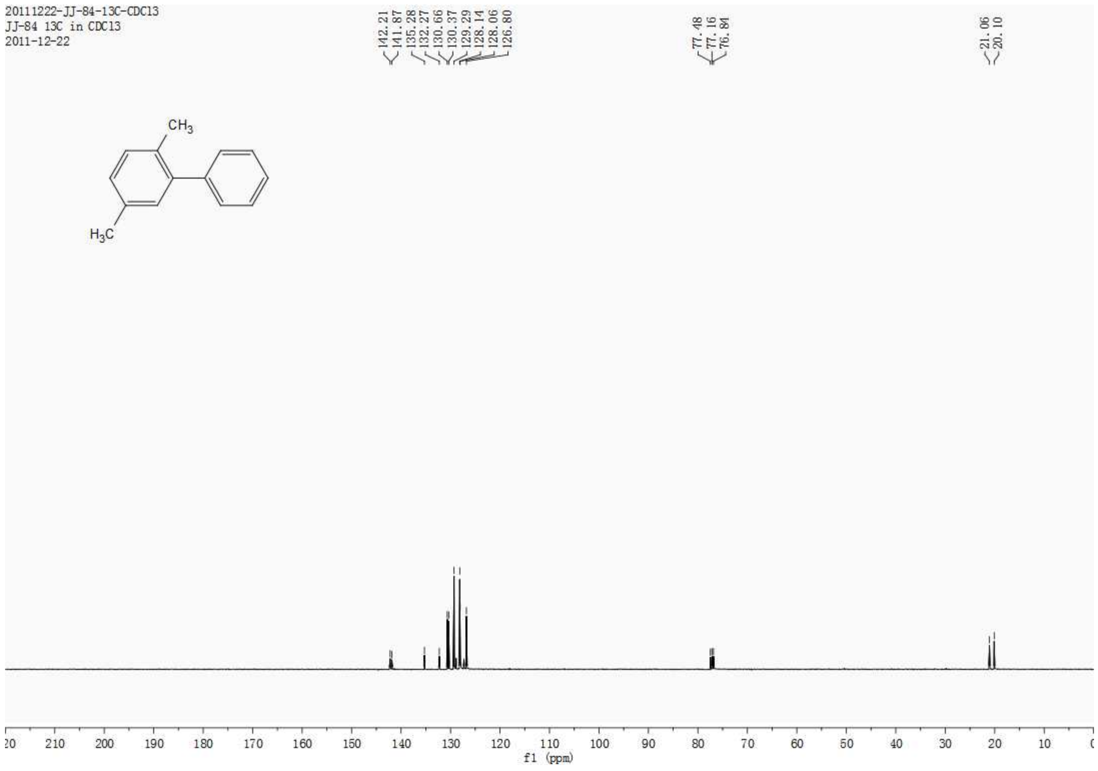

Figure S25. ^{1}H-NMR spectrum of **12m**.

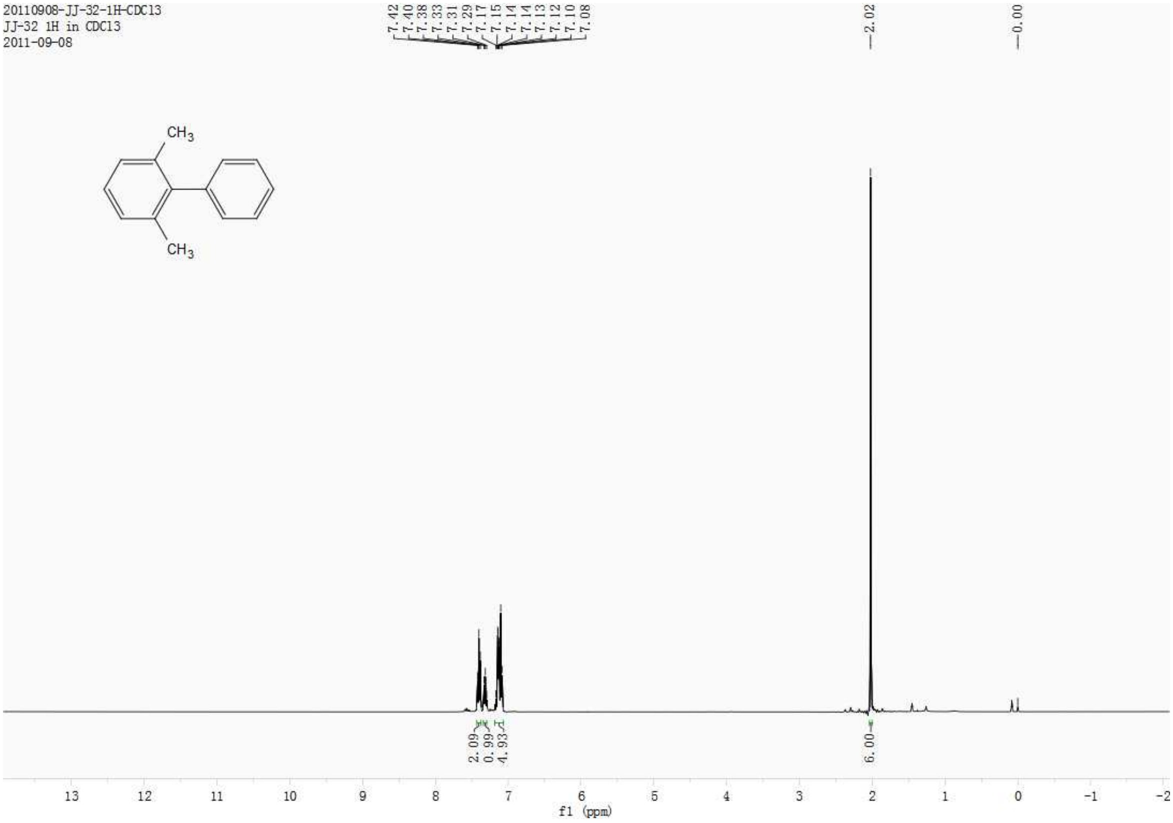

Figure S26. ^{13}C-NMR spectrum of **12m**.

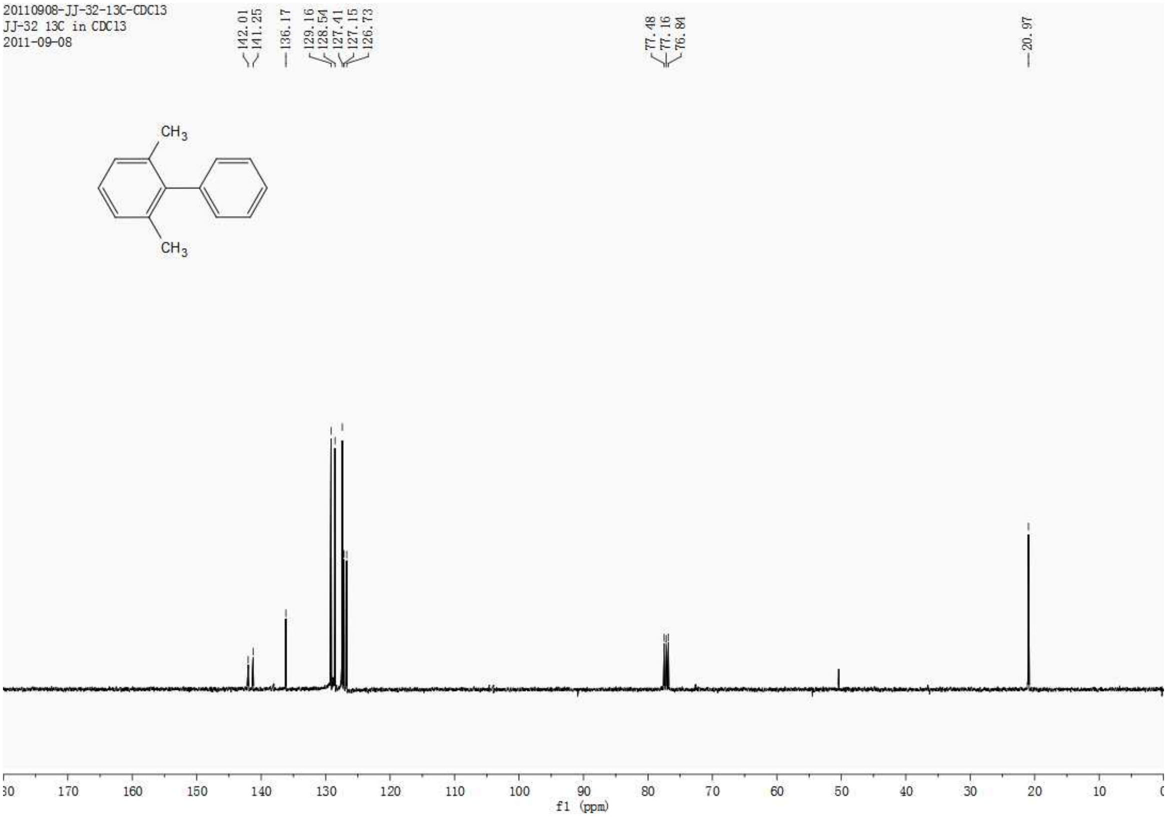

Figure S27. ^{1}H-NMR spectrum of **12n**.

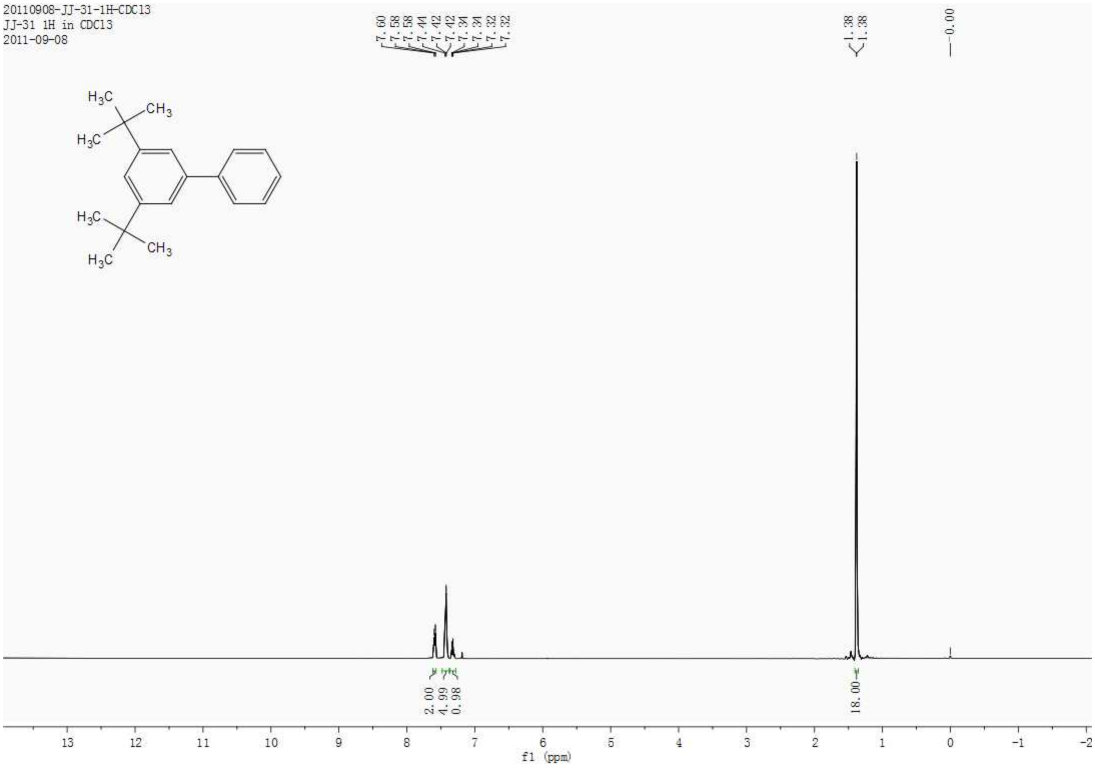

Figure S28. ^{13}C-NMR spectrum of **12n**.

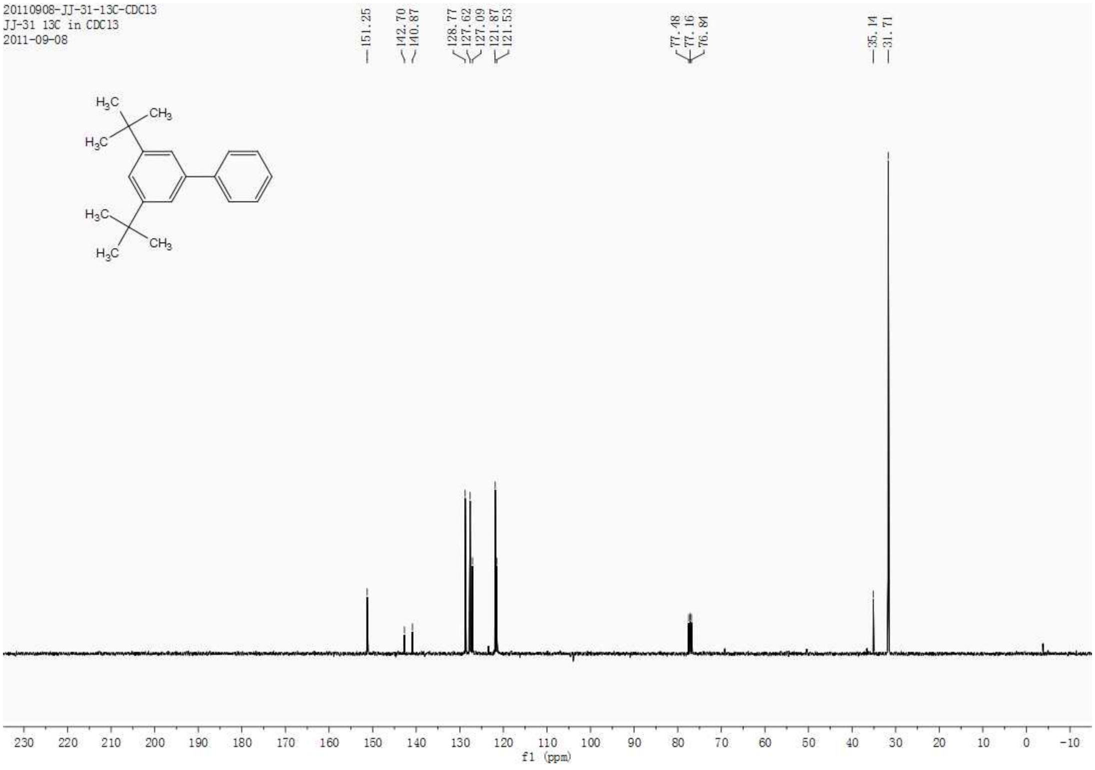

Figure S29. ^{1}H-NMR spectrum of **12o**.

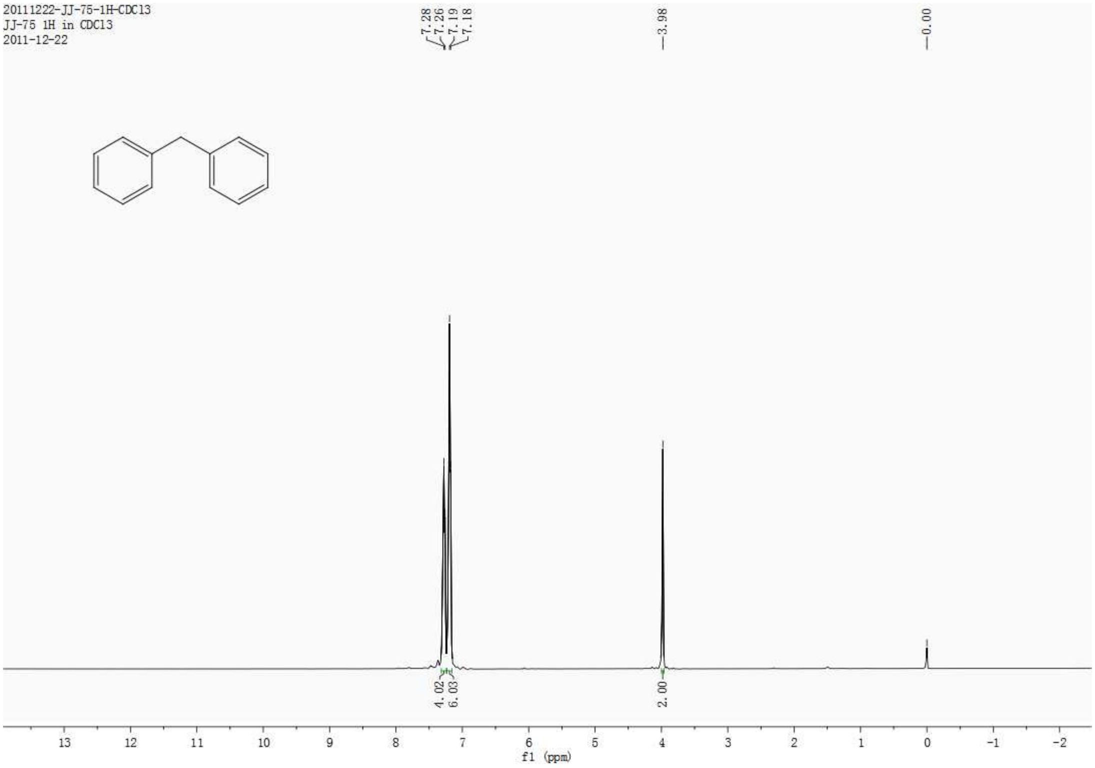

Figure S30. ^{13}C-NMR spectrum of **12o**.

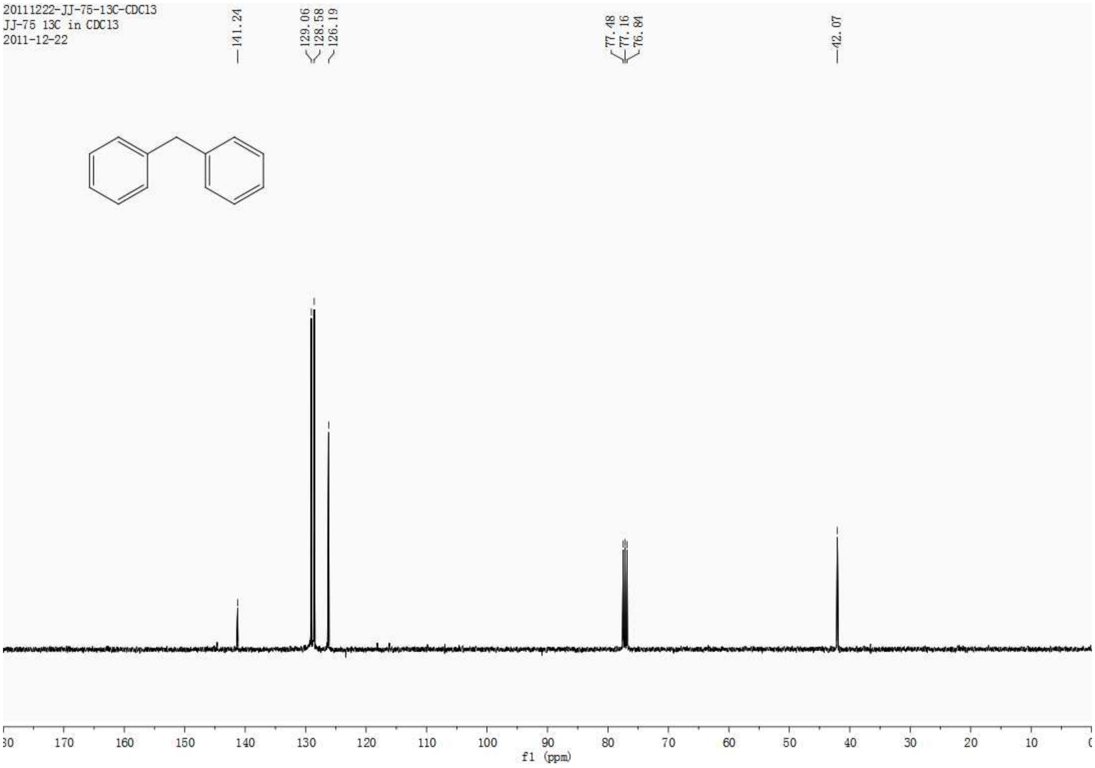

Figure S31. ^{1}H-NMR spectrum of **12p**.

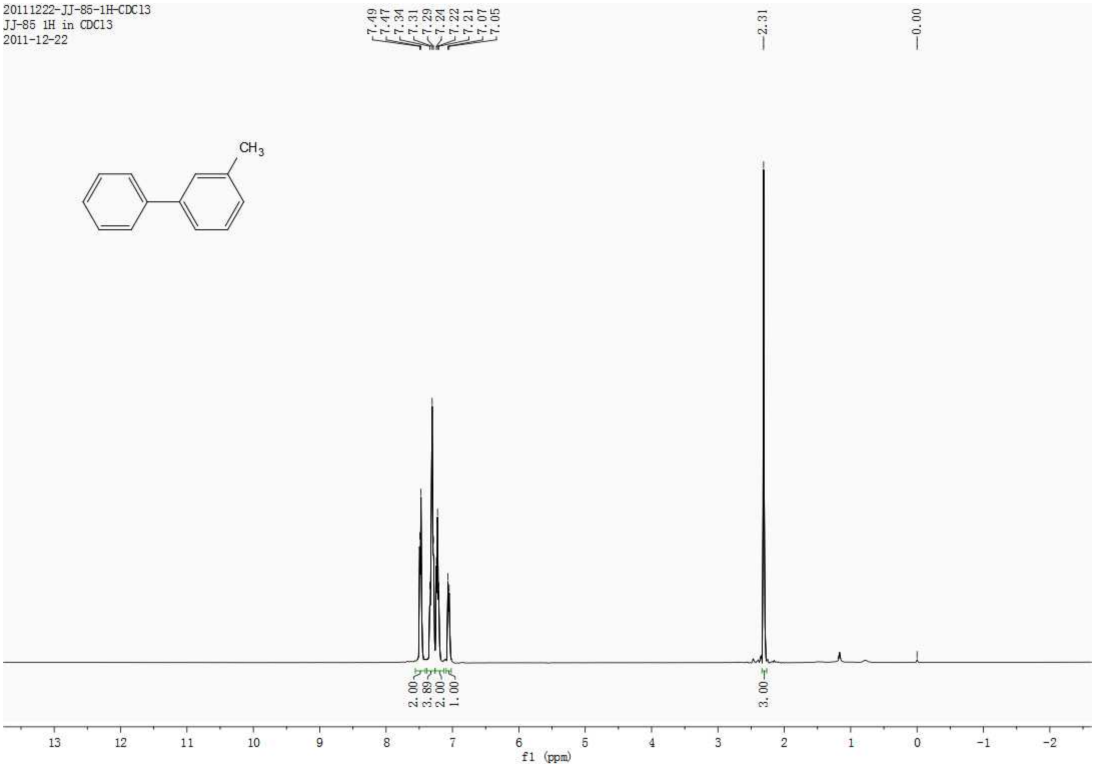

Figure S32. ^{13}C-NMR spectrum of **12p**.

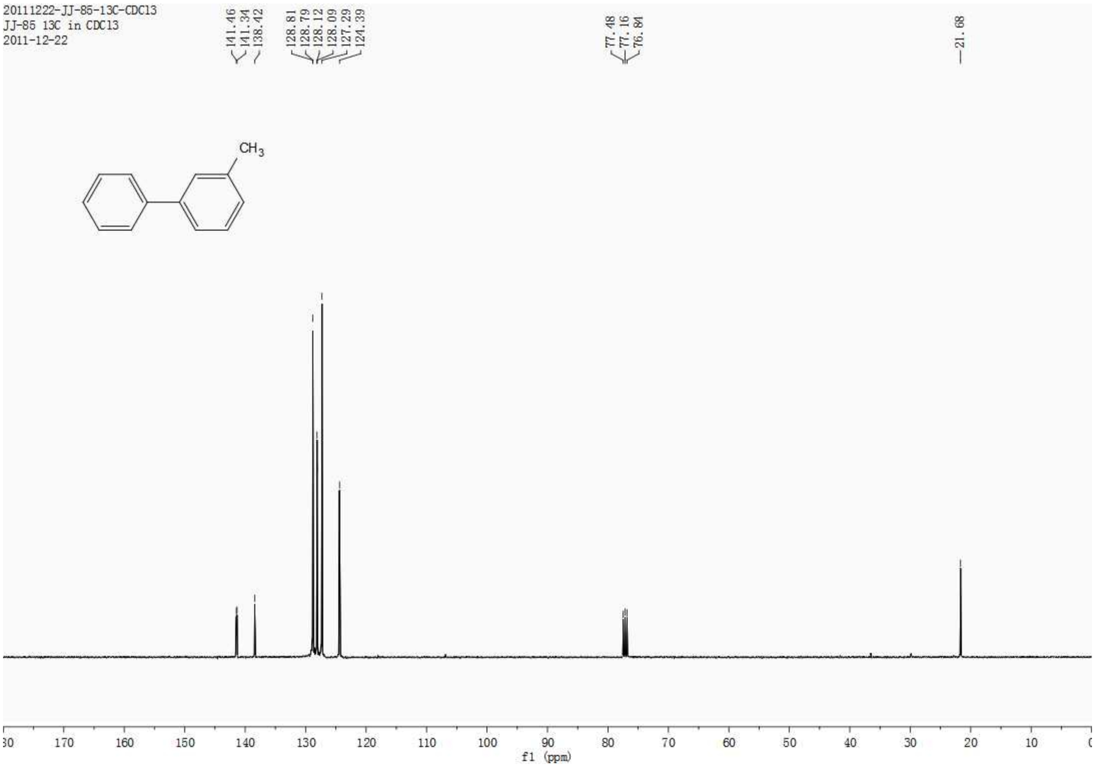

Figure S33. ^{1}H-NMR spectrum of **12q**.

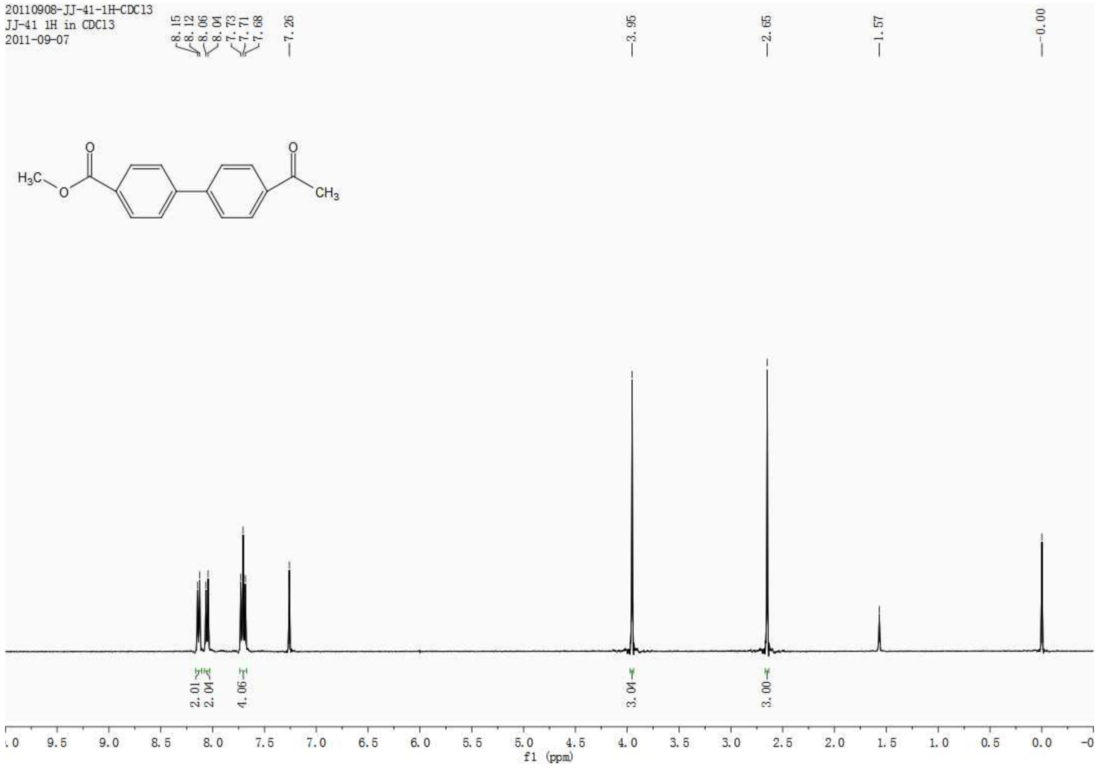

Figure S34. ^{13}C-NMR spectrum of **12q**.

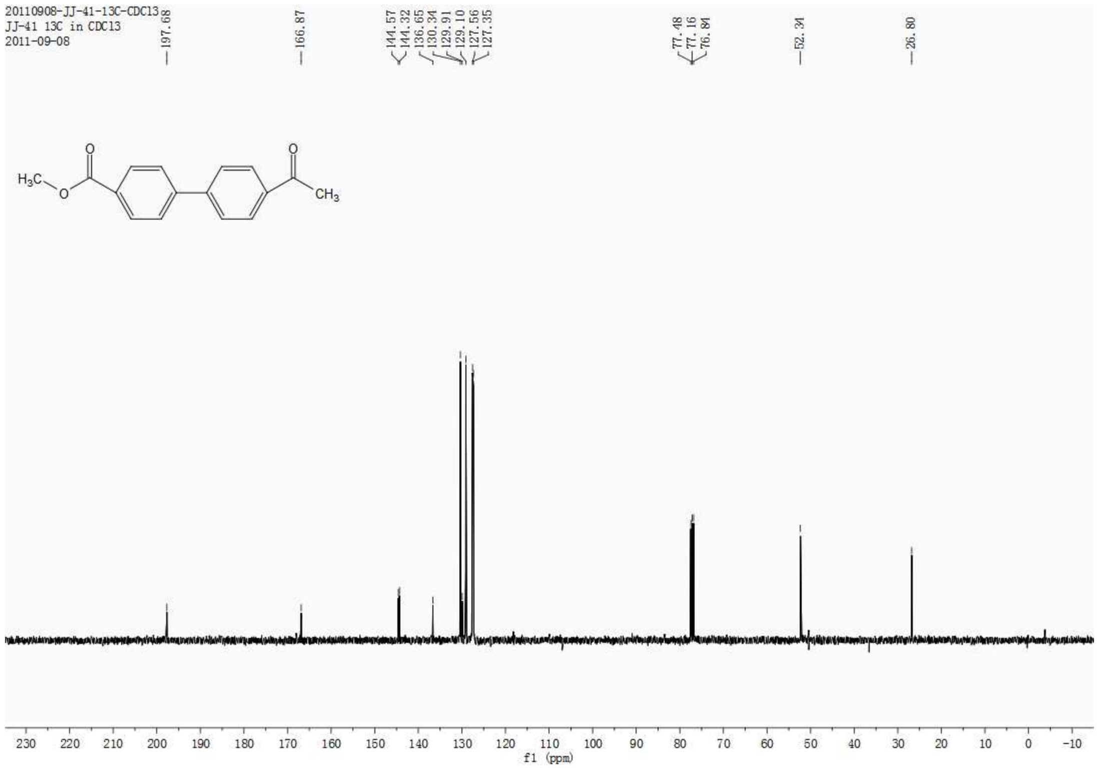

2. ^{1}H-NMR and ^{13}C-NMR spectra of 8f–11f and the imidazolium salts 7a–f

Figure S35. ^{1}H-NMR spectrum of **8f**.

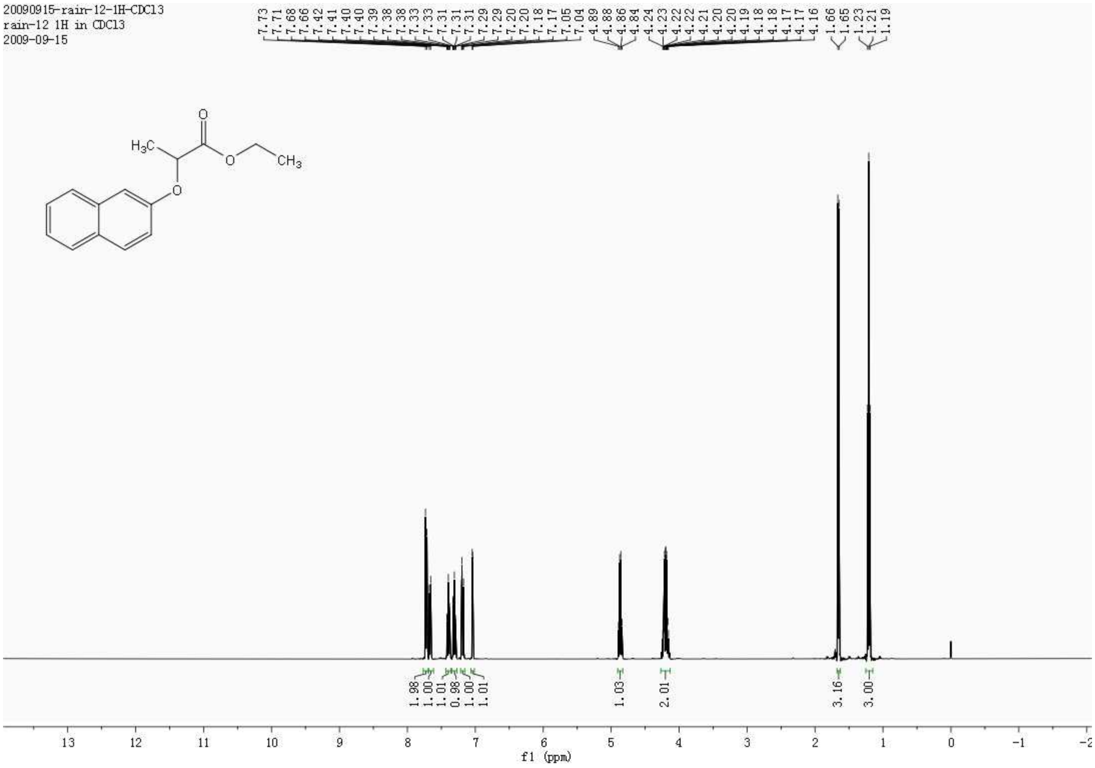

Figure S36. ^{13}C-NMR spectrum of **8f**.

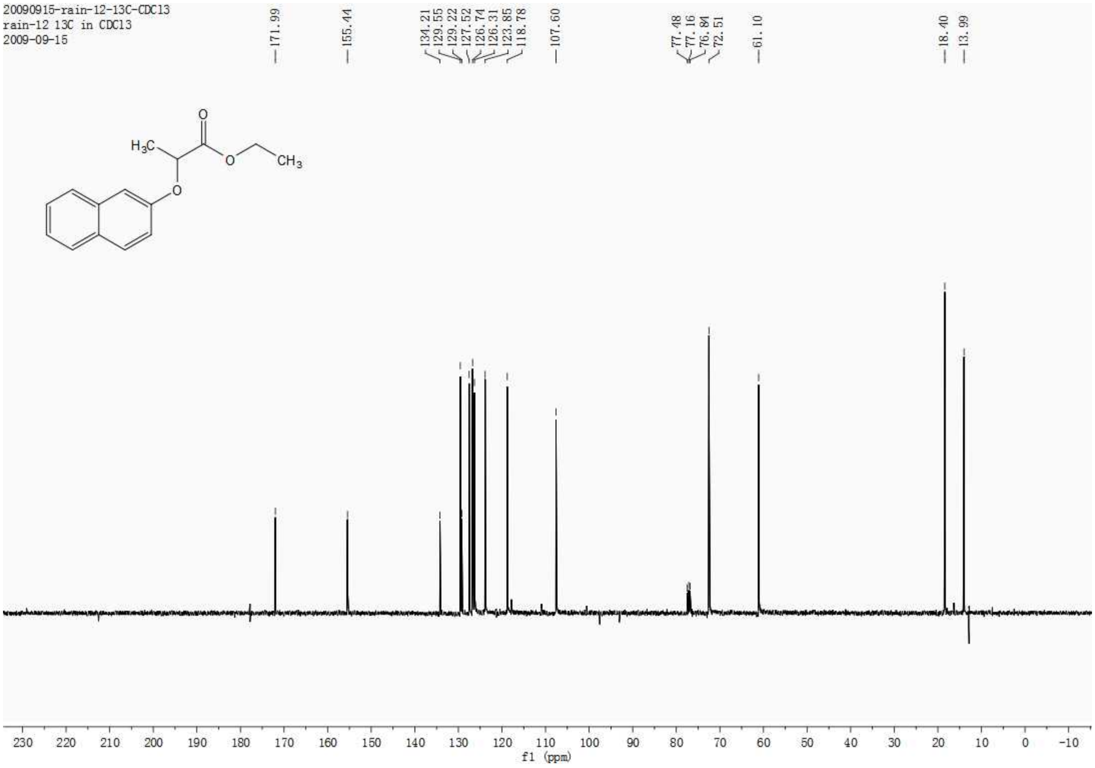

Figure S37. ^{1}H-NMR spectrum of **9f**.

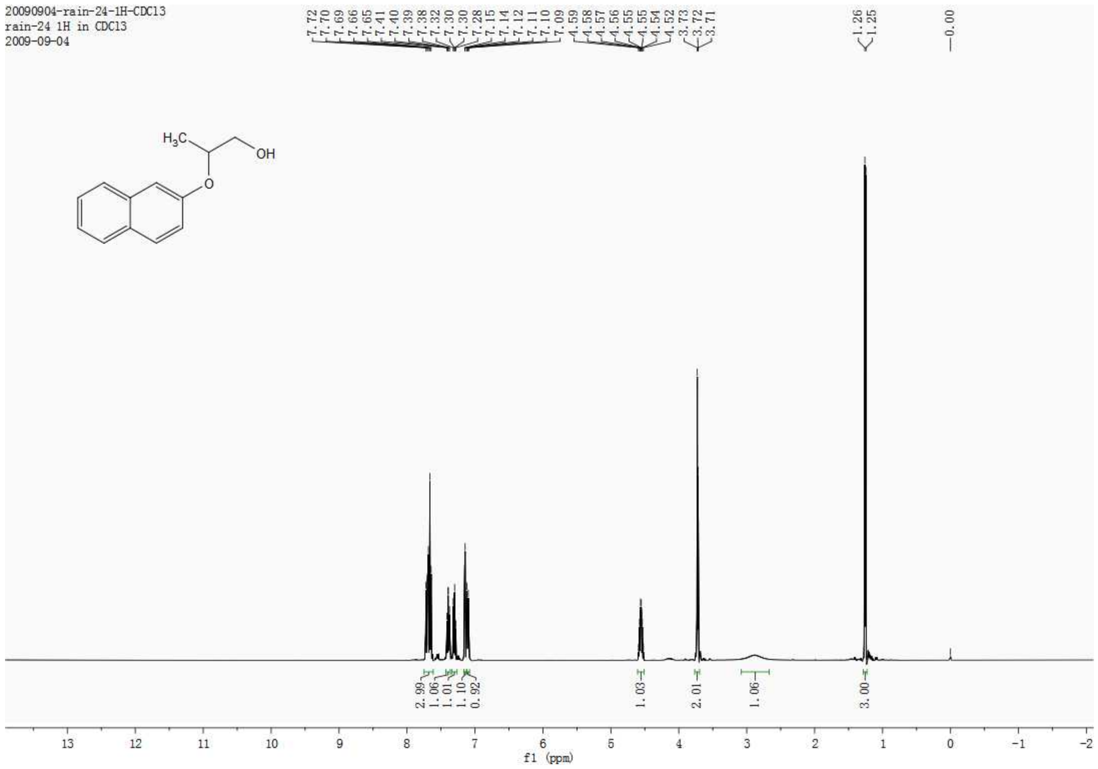

Figure S38. ^{13}C-NMR spectrum of **9f**.

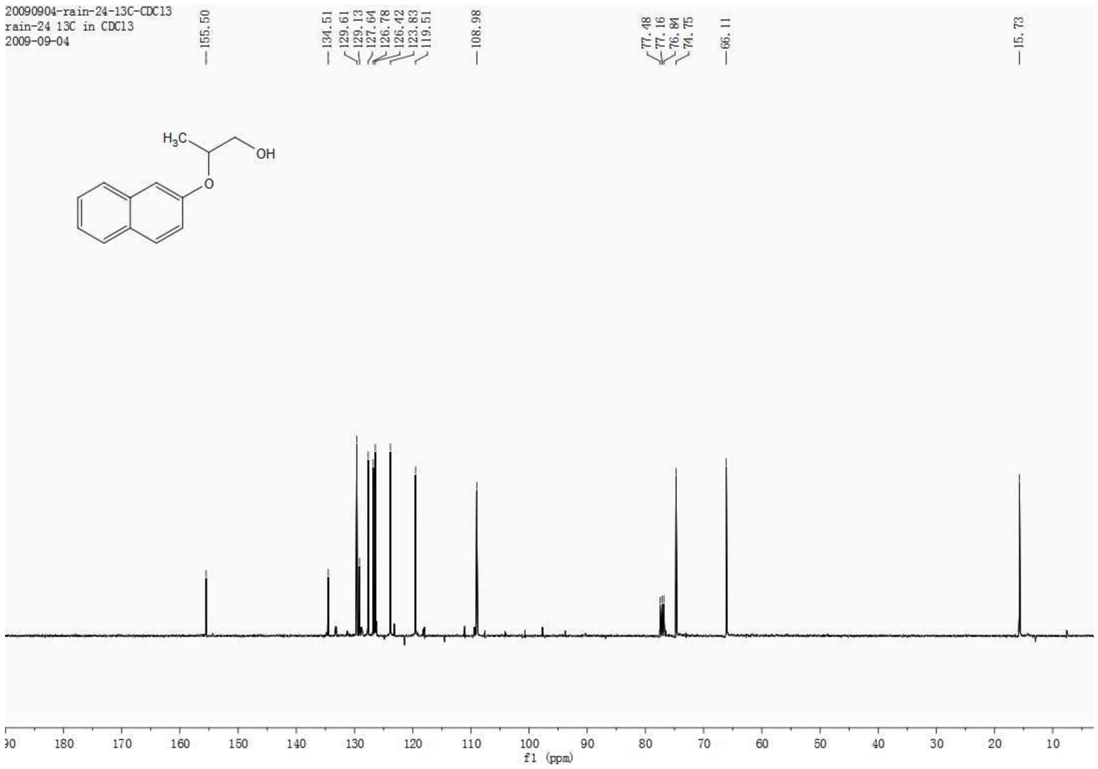

Figure S39. ^{1}H-NMR spectrum of **10f**.

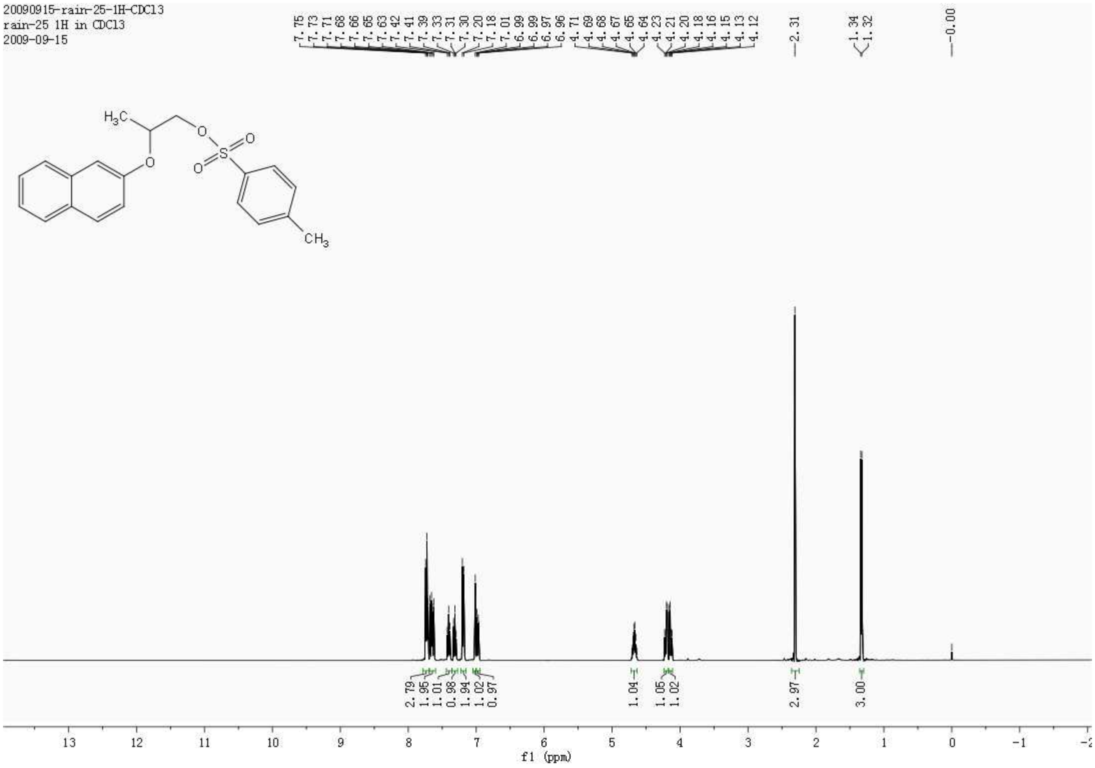

Figure S40. ^{13}C-NMR spectrum of **10f**.

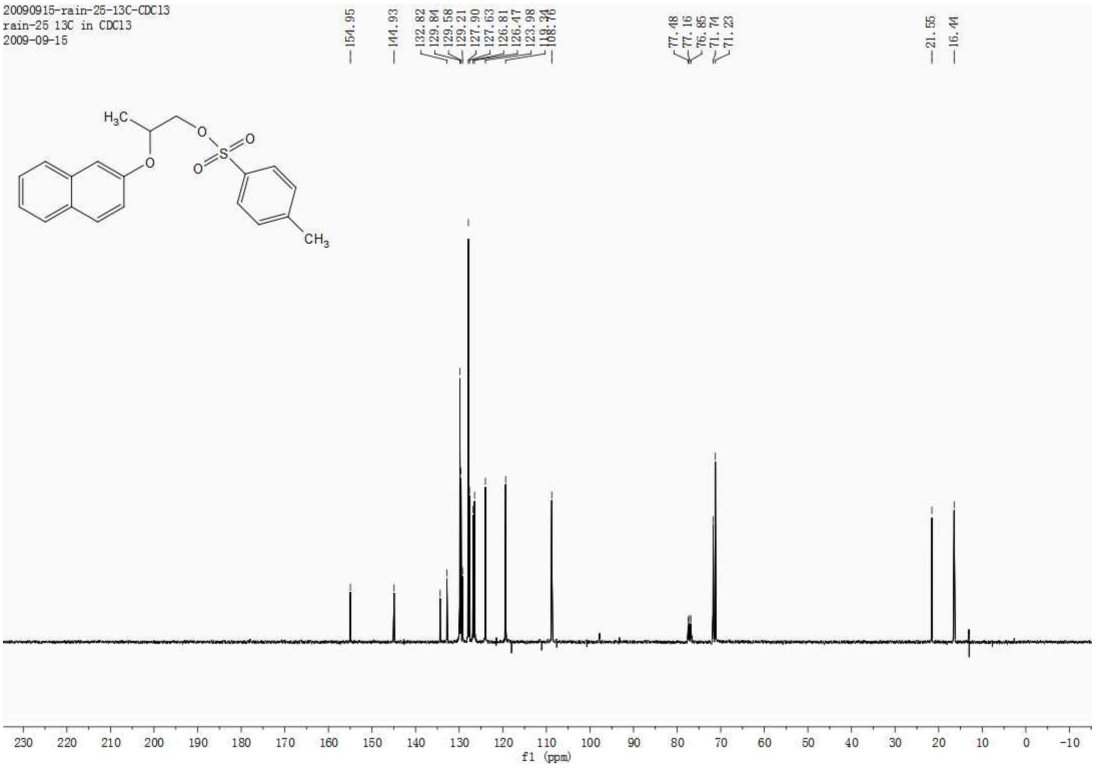

Figure S41. ^{1}H-NMR spectrum of **11f**.

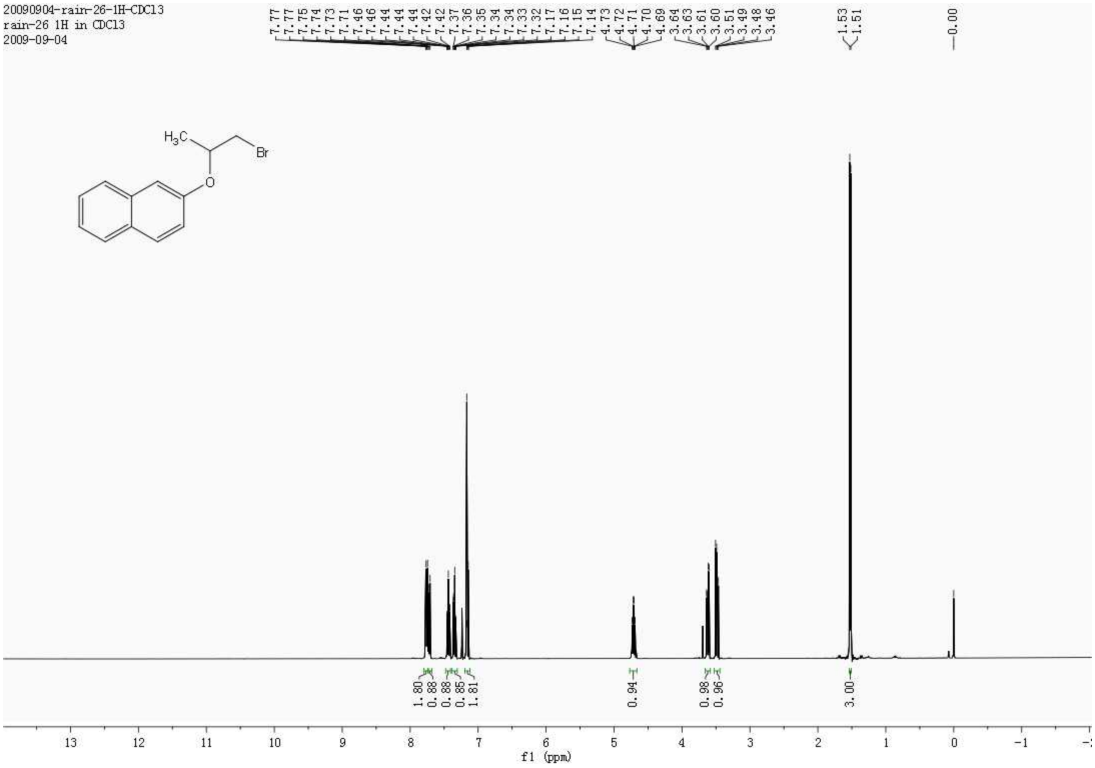

Figure S42. ^{13}C-NMR spectrum of **11f**.

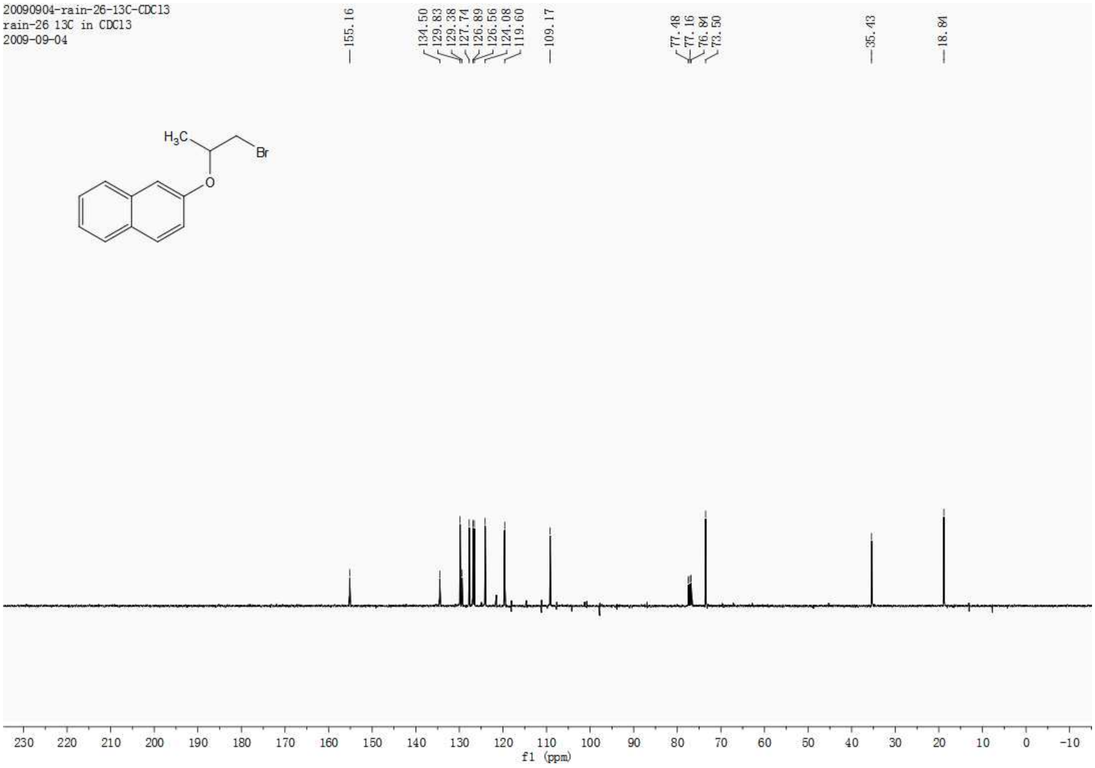

Figure S43. ^{1}H-NMR spectrum of **7a**.

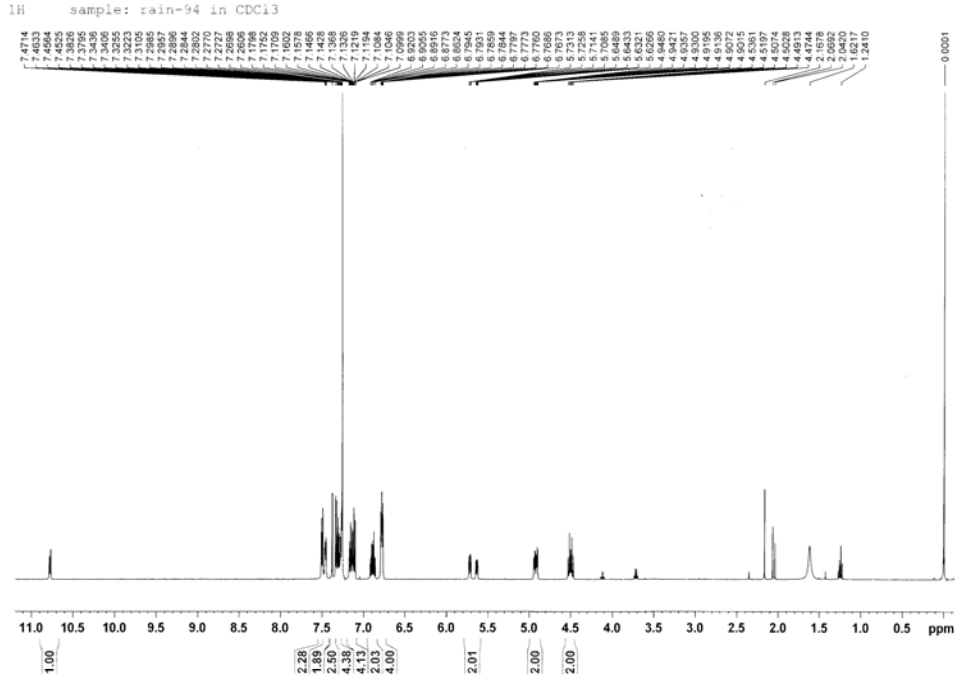

Figure S44. ^{13}C-NMR spectrum of **7a**.

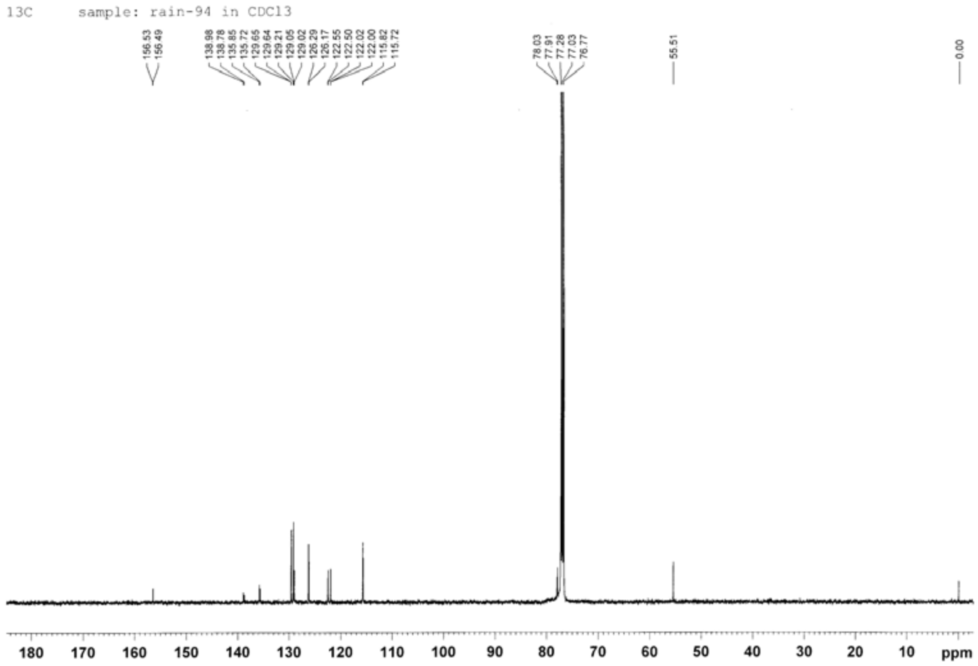

Figure S45. ^{1}H-NMR spectrum of **7b**.

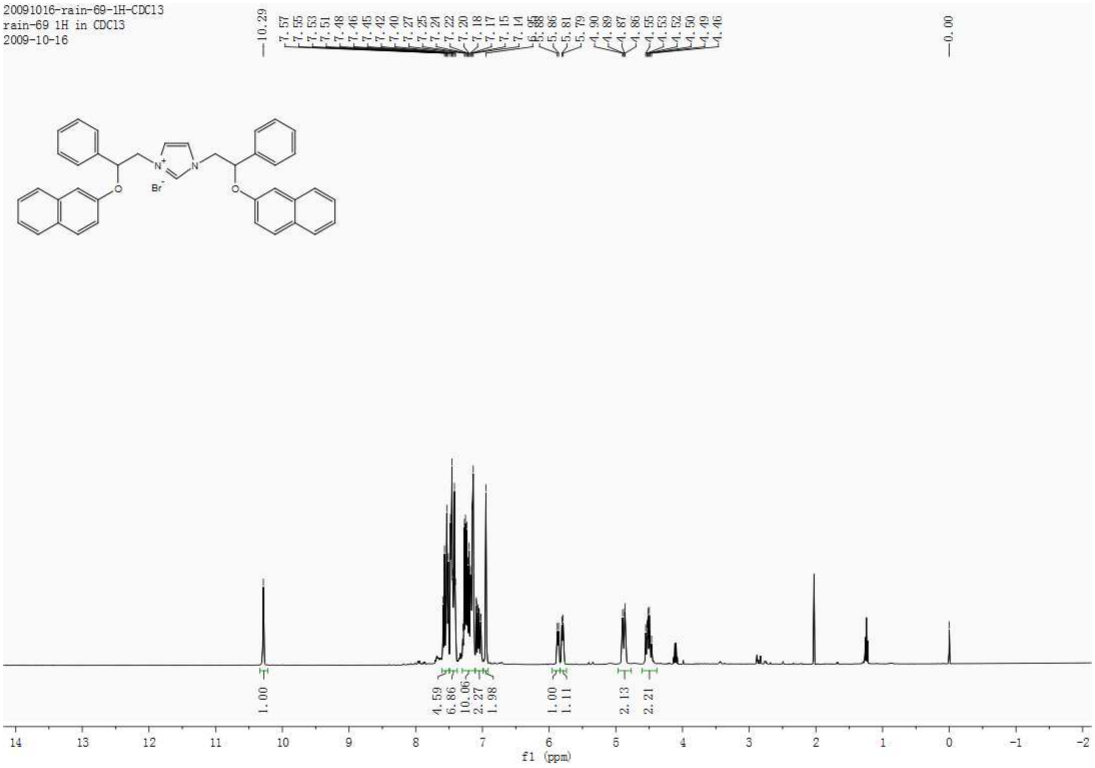

Figure S46. ^{13}C-NMR spectrum of **7b**.

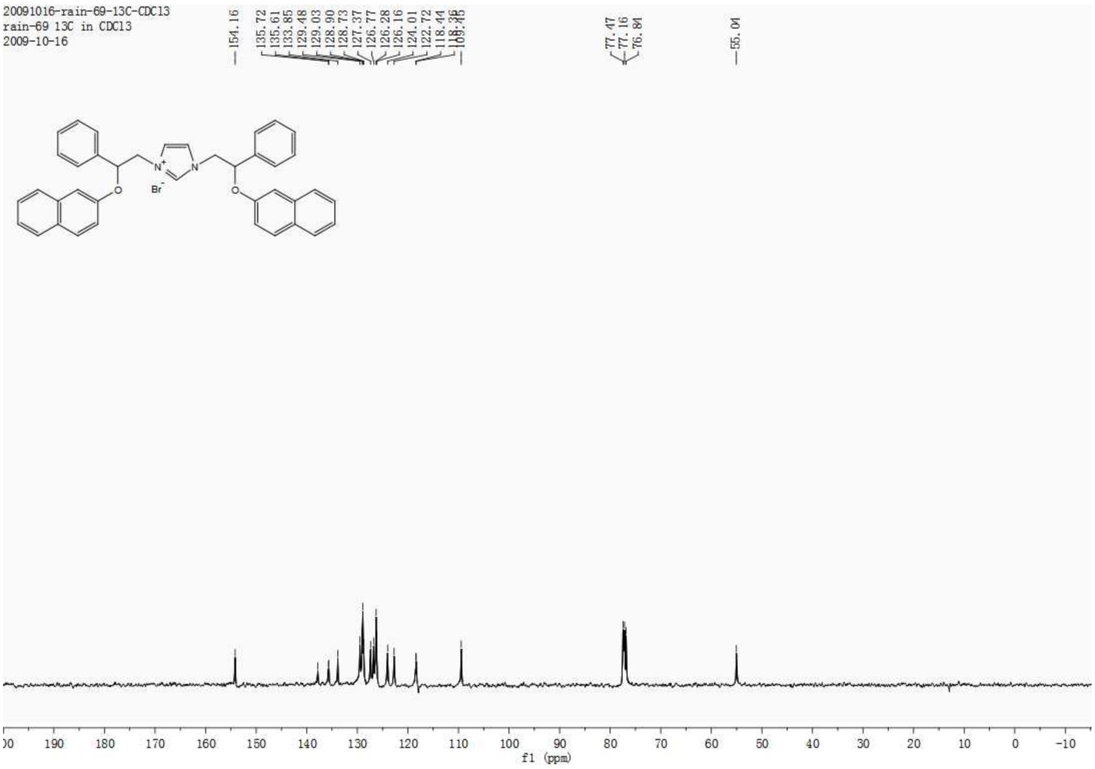

Figure S47. ^{1}H-NMR spectrum of **7c**.

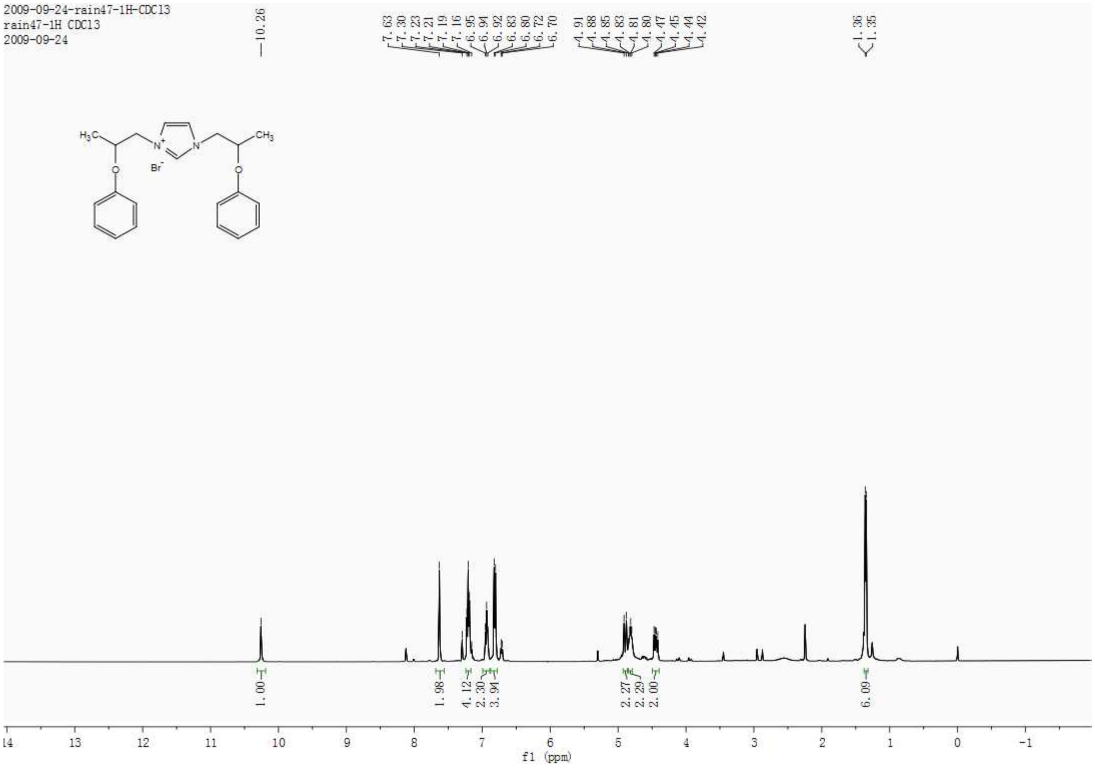

Figure S48. ^{13}C-NMR spectrum of **7c**.

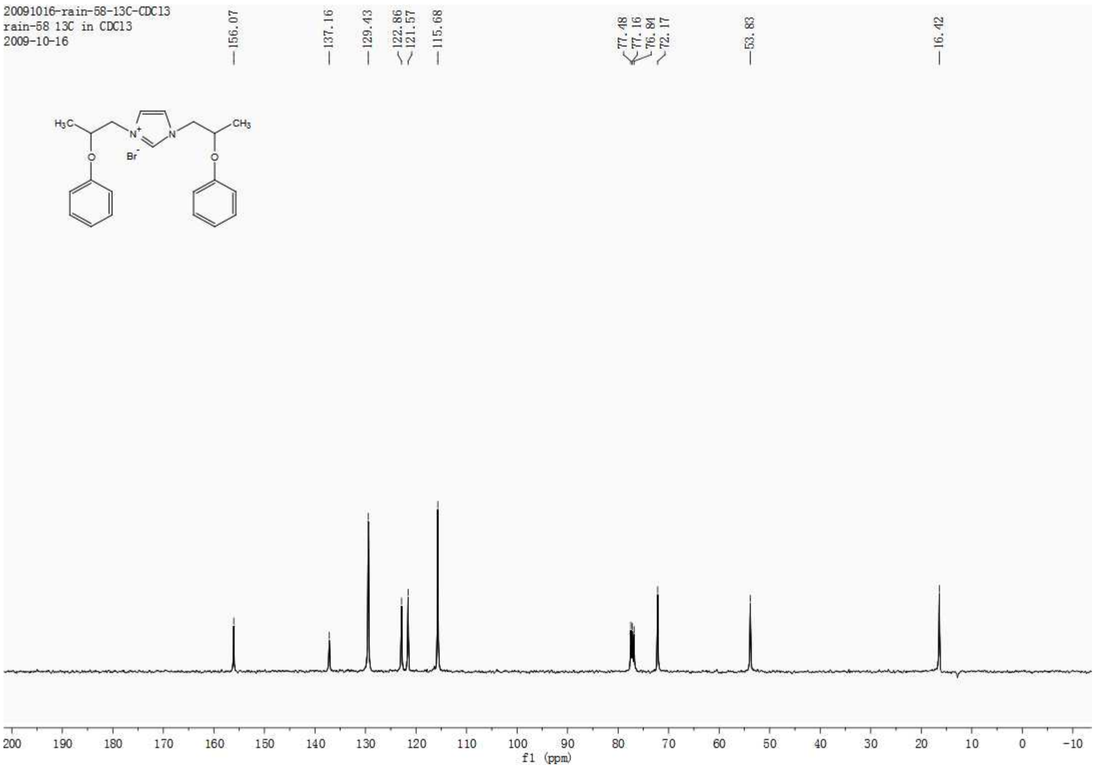

Figure S49. ^{1}H-NMR spectrum of **7d**.

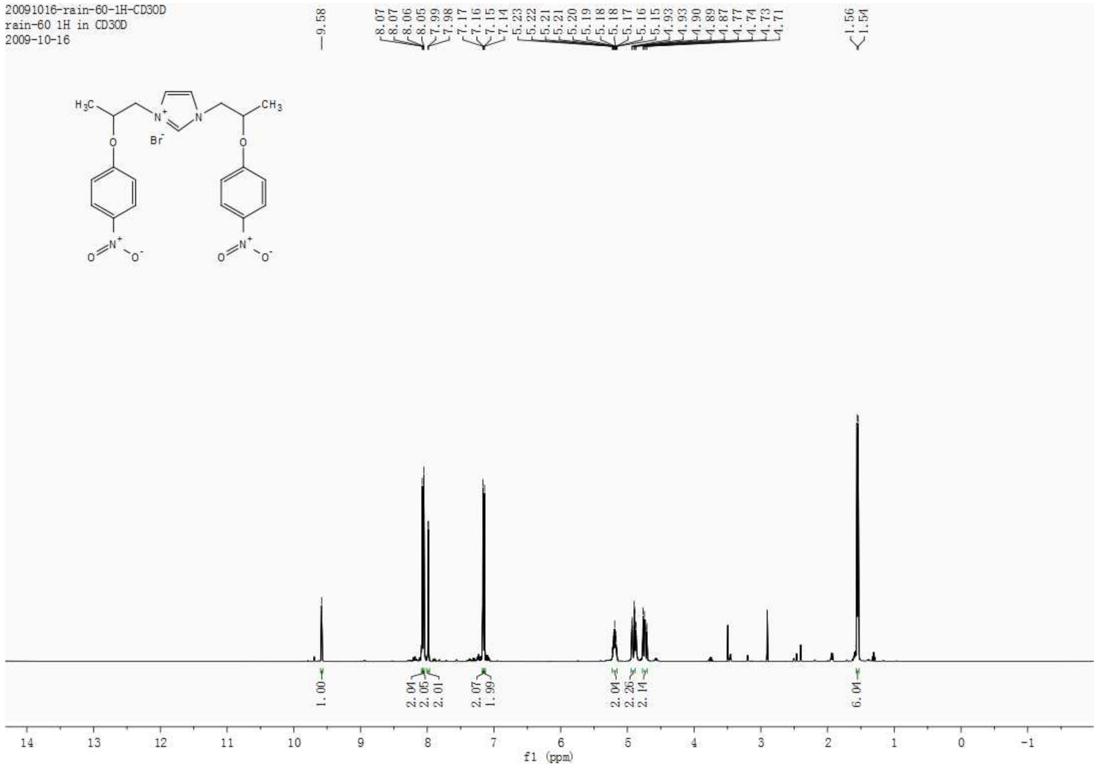

Figure S50. ^{13}C-NMR spectrum of **7d**.

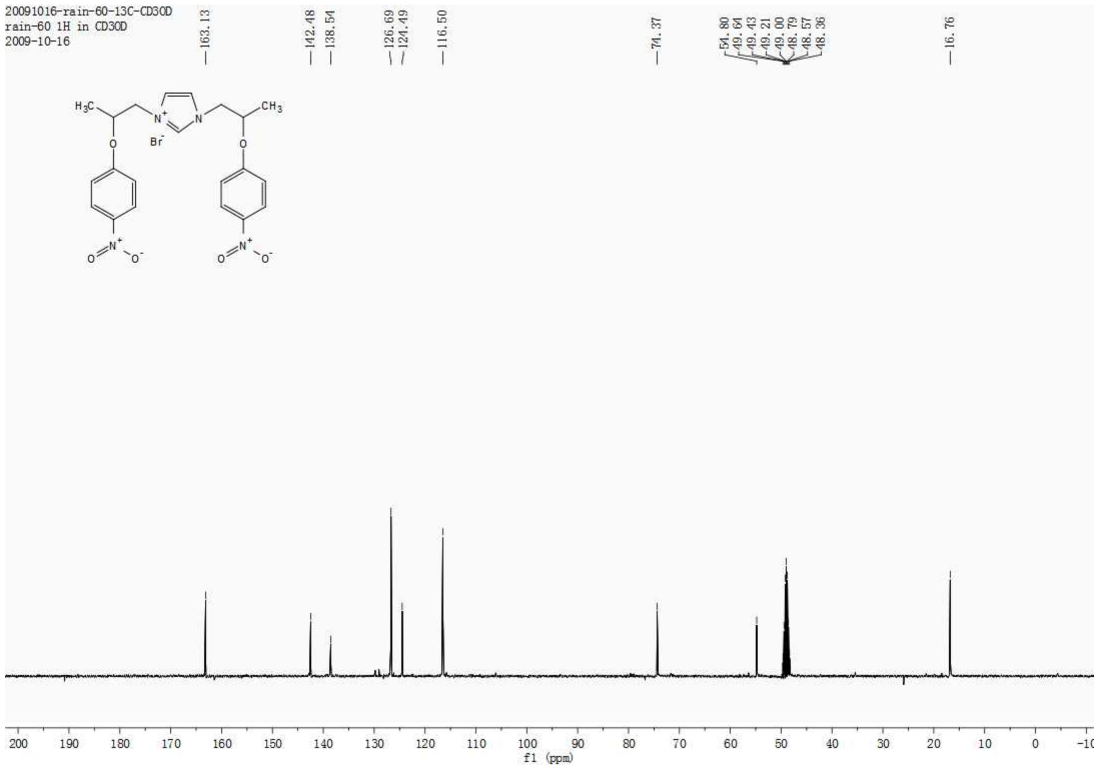

Figure S51. ^{1}H-NMR spectrum of **7e**.

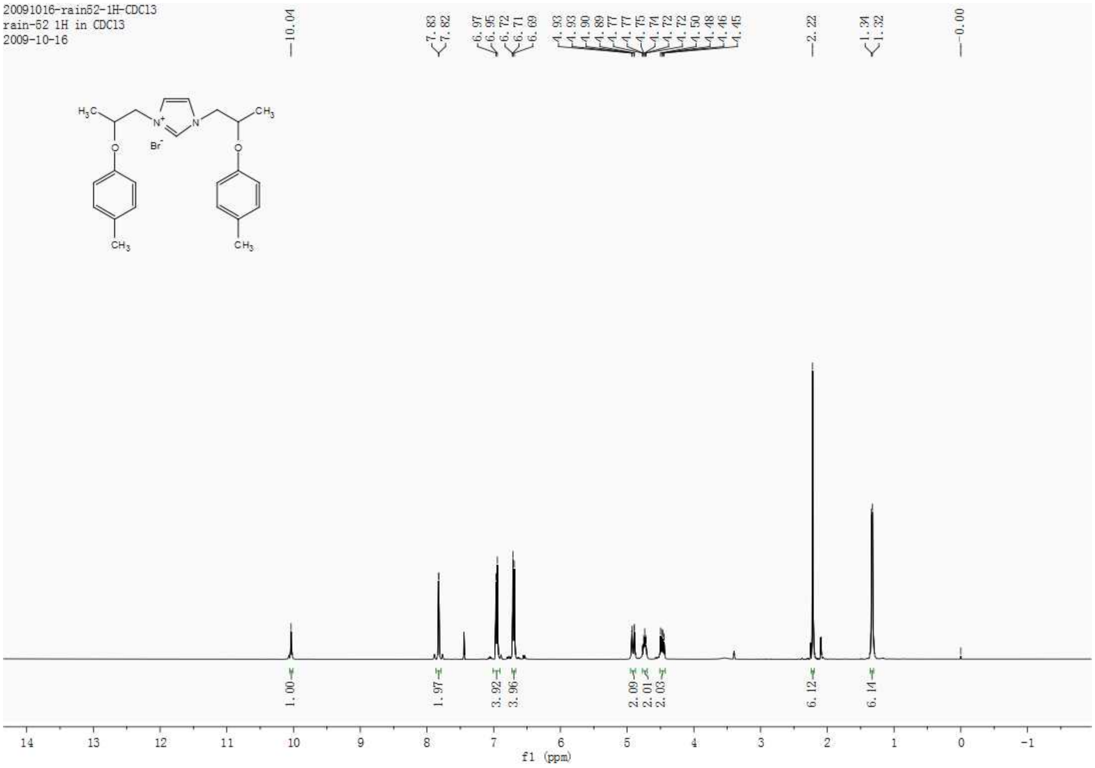

Figure S52. ^{13}C-NMR spectrum of **7e**.

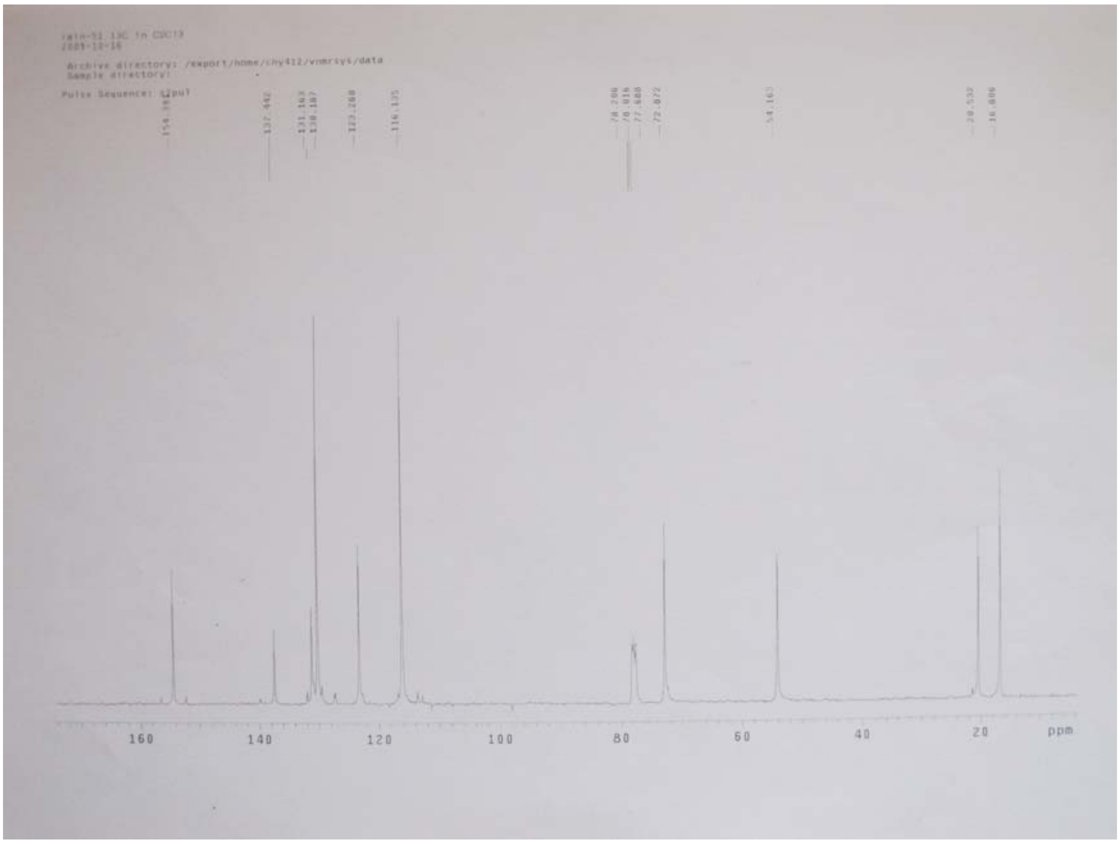

Figure S53. ^{1}H-NMR spectrum of **7f**.

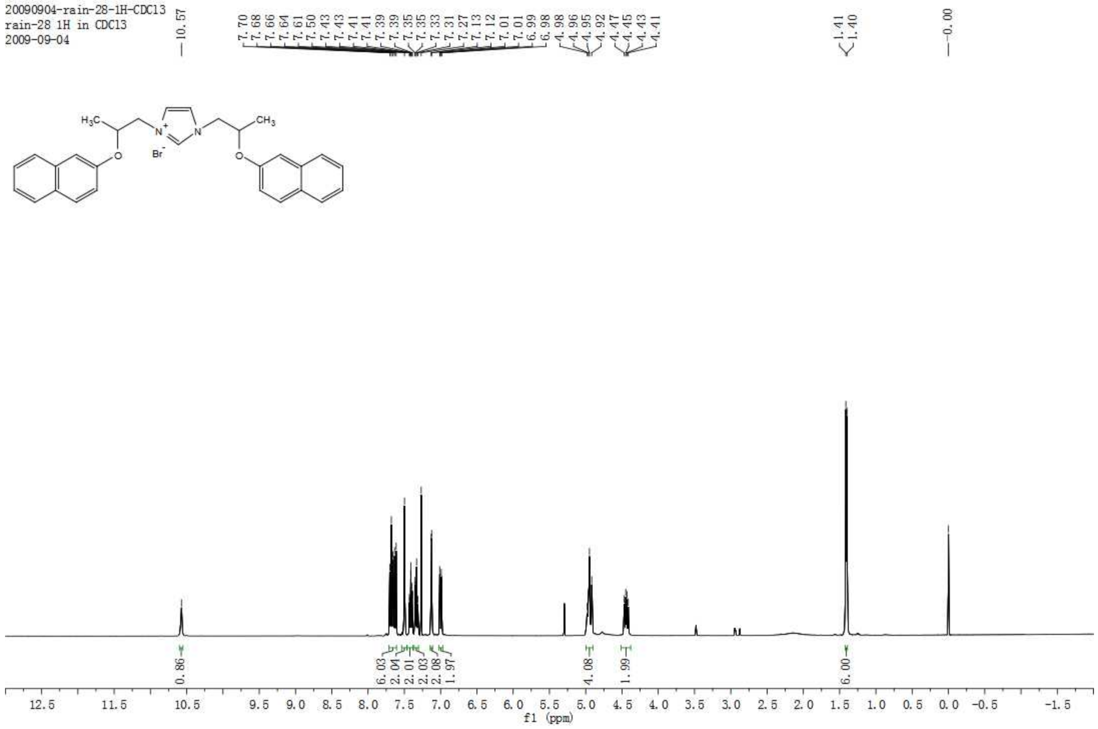

Figure S54. ^{13}C-NMR spectrum of **7f**.

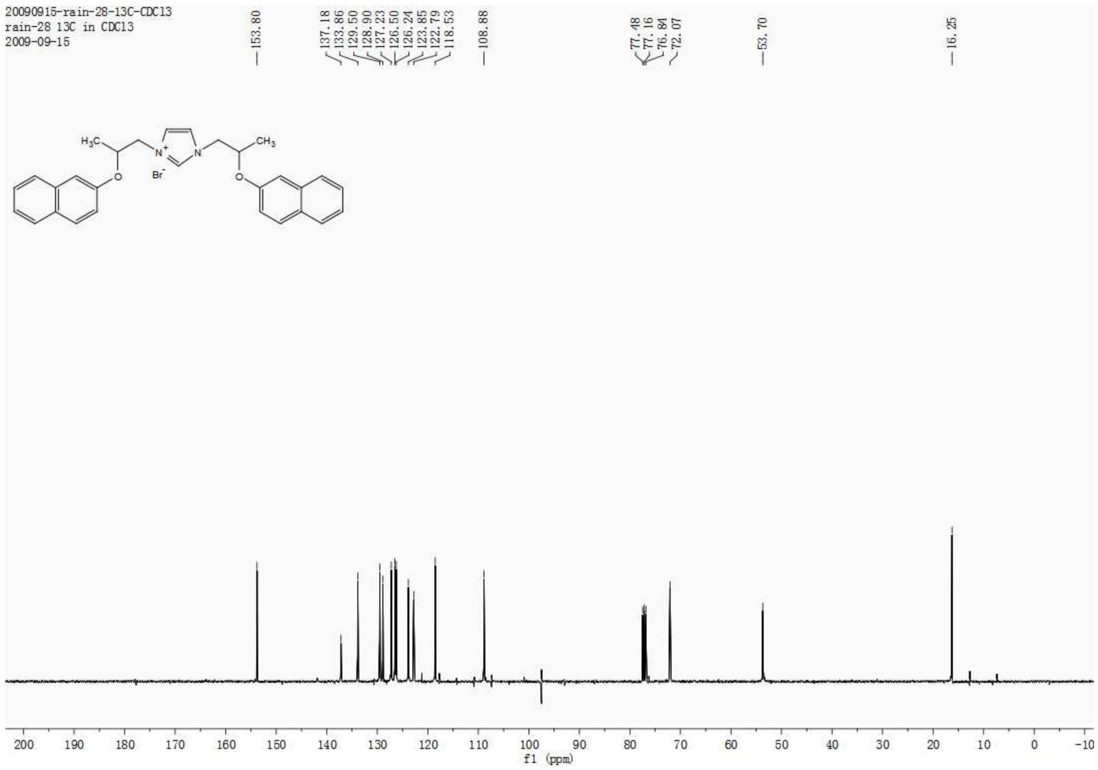

Supplement: Supplementary file 1 [file molecules-17-12121-s001.pdf]
